# Light‐Gated Amine Exchange in Diarylethene‐Crosslinked Microgels

**DOI:** 10.1002/anie.202510141

**Published:** 2025-08-22

**Authors:** Kevin Broi, Frédéric Grabowski, Sarah Esser, Jannick Dörr, Andrij Pich, Stefan Hecht

**Affiliations:** ^1^ Institute of Technical and Macromolecular Chemistry RWTH Aachen University Worringerweg 2 52074 Aachen Germany; ^2^ DWI–Leibniz Institute for Interactive Materials RWTH Aachen University Forckenbeckstr. 50 52074 Aachen Germany; ^3^ Aachen Maastricht Institute for Biobased Materials Maastricht University Geleen 6167 RD The Netherlands; ^4^ Department of Chemistry, Humboldt University of Berlin Brook‐Taylor‐Str. 2 12489 Berlin Germany; ^5^ Center for the Science of Materials Berlin Humboldt University of Berlin Zum Großen Windkanal 2 12489 Berlin Germany

**Keywords:** Colloids, Diarylethenes, Drug delivery, Dynamic covalent chemistry, Switchable microgels

## Abstract

The functionalization of microgels with photoswitchable moieties enables the construction of multi‐responsive systems, which are highly interesting for biological applications, for example, in drug delivery. Controlling the reversible uptake of functional molecules, however, remains a major challenge. To address this, we developed a novel aniline‐type diarylethene (DAE) crosslinker and prepared a series of corresponding poly(*N*‐vinylcaprolactam)‐based microgels with varying degrees of DAE crosslinker. Upon UV light irradiation, the aniline (and thus enamine) motif of the DAE is converted to its imine form, thus allowing for reversible exchange with primary amines. Irradiating the DAE with blue light covalently locks the equilibrium and traps the amine in the microgels. The light‐controlled covalent binding of fluorinated, hydrophobic, and hydrophilic amine derivatives was proven by dynamic light scattering and ^19^F NMR spectroscopy. Importantly, the impact of photoisomerization and amine binding on microgel properties was investigated and we were able to remotely control size as well as temperature and pH responsiveness by light. Overall, this study demonstrates optically gated uptake and release of functional amines in multi‐responsive microgels and highlights their potential biological applications.

## Graphical abstract

Integrating aniline‐type diarylethenes as core‐enriched crosslinkers in poly(*N*‐vinylcaprolactam) based microgels enables photomodulation of their responsivity towards temperature and pH as well as covalent binding and release of functional amines via light‐gated amine exchange reaction. This study highlights the potential of photoswitchable microgels for smart biomedical materials.

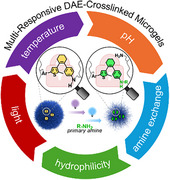

## Introduction

Controlling the size and molecular architecture in materials is of crucial importance to localize functionalities down to the nanometer scale. In recent years, flexible and scalable polymerization methods have been developed to obtain functional materials and in particular microgels have emerged as an attractive material class providing a unique and scalable means for compartmentalization, that is, specific positioning of functionalities.^[^ ^1^, ^2^ ^]^ Microgels are stimuli‐responsive soft materials consisting of a three‐dimensional crosslinked polymer network structure and have found applications in a wide range of areas from catalysis,^[^ ^3^, ^4^ ^]^ sensing,^[^ ^5^, ^6^ ^]^ coating,^[^ ^7^, ^8^ ^]^ and tissue engineering^[^ ^9^, ^10^ ^]^ to drug delivery.^[^ ^11^, ^12^ ^]^ Part of this versatility comes from the fact that microgels are highly biocompatible and are swollen in a solvent maintaining their colloidal stability.^[^ ^13^, ^14^ ^]^ In this context, poly(*N*‐vinylcaprolactam) (PVCL) based microgels exhibit a volume phase transition temperature (VPTT) of around 32 °C in water.^[^ ^14^, ^15^ ^]^ Therefore, microgels are also referred to as “smart” materials, whereby the behavior can be further tuned by additional modification with specific molecular switches to respond to other external stimuli such as pH,^[^ ^16^, ^17^ ^]^ magnetic fields,^[^ ^18^, ^19^ ^]^ or light.^[^ ^20^, ^21^, ^22^, ^23^, ^24^, ^25^ ^]^

Light as a non‐invasive and environmentally friendly stimulus combines excellent temporal and spatial resolution with the ability to reversibly activate/bind certain molecular entities to control specific material properties such as (self‐)healing or adhesion.^[^ ^26^, ^27^, ^28^, ^29^ ^]^ Molecular photoswitches therefore serve as an attractive platform to integrate an optical response into microgels and allow controlling, for example, color/absorption, polarity, size, shape, and geometry for potential applications.^[^ ^21^, ^22^, ^23^, ^24^, ^30^, ^31^ ^]^ For instance, by incorporation of thermally reversible, that is, T‐type, photoswitches such as spiropyrans^[^ ^32^, ^33^ ^]^ or donor‐acceptor Stenhouse adducts (DASAs)^[^ ^34^, ^35^ ^]^ in the shell of PVCL‐based microgels, substantial changes of the polarity could be achieved by illuminating with UV as well as visible light. Light‐induced isomerization of the switch increased hydrophilic interactions that led to swelling.^[^ ^21^, ^24^ ^]^ Another approach made use of acrylic acid and an azobenzene^[^ ^36^, ^37^, ^38^ ^]^ photoswitch in the microgel shell to achieve light‐ and pH‐responsive swelling.^[^ ^22^ ^]^ However, due to the thermally reversible nature of the azobenzene, clean switching between an “ON” and “OFF” state exquisitely by light is impossible. Moreover, incorporation requires either the utilization of (co‐)polymerizable groups or certain post‐functionalization techniques, which can further limit synthetic flexibility. In contrast, P‐type photochromic diarylethenes (DAEs) can overcome both of these limitations due to their thermal bistability as well as highly versatile modular synthesis.^[^ ^39^, ^40^ ^]^ In this regard, a more recent approach utilized a bivalent methacrylate‐functionalized DAE derivative as a co‐crosslinker for PVCL‐based microgels to shift the VPTT to lower values upon UV light irradiation. Here, the accompanying 6π‐electrocyclization of the cross‐conjugated ring‐open DAE core motif to its less polar, more stiff, and fully π‐conjugated ring‐closed isomer leads to more pronounced hydrophobic interactions, explaining the observed VPTT shift.^[^ ^23^ ^]^ With respect to drug delivery, however, controlling microscopic reactivity, for example, covalent bond formation and scission, becomes increasingly relevant with regard to the reversible uptake of bioactive molecules. In this context, dynamic covalent chemistry^[^ ^41^ ^]^ serves as one of the most prominent tools to achieve such light‐gated reactivity, which has been demonstrated for DAEs.^[^ ^42^, ^43^ ^]^ In particular, the exchange of amines – omnipresent in Nature – under ambient conditions constitutes an attractive target reaction^[^ ^29^, ^44^, ^45^, ^46^, ^47^, ^48^, ^49^ ^]^ and thus light‐gating their covalent trapping and release within a microgel environment should prove highly beneficial in the context of life science applications.

**Figure 1:**
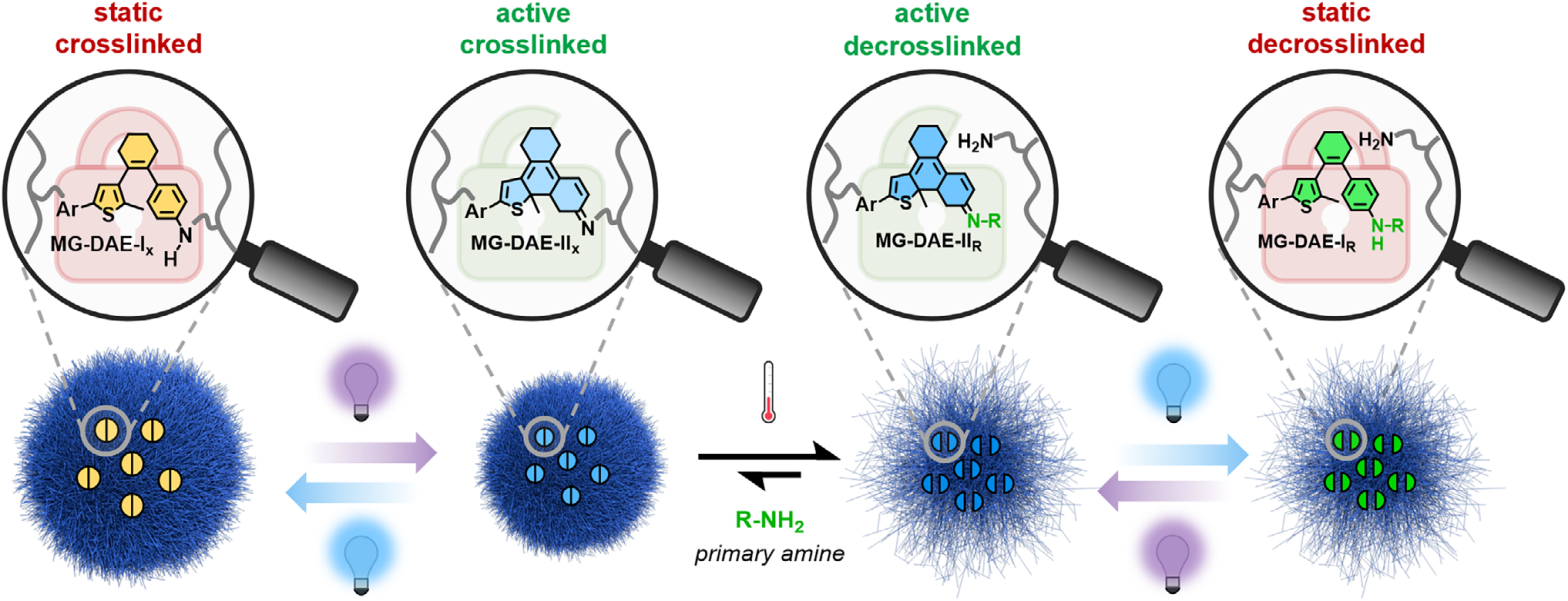
Light‐gated post‐modification of DAE‐crosslinked PVCL‐based microgels: UV light induced ring‐closure of **MG‐DAE‐I_x_** leads to the formation of active imine **MG‐DAE‐II_x_**, causing a shrinkage of the microgel due a reduced flexibility of the more rigid ring‐closed DAE structure. Treatment with primary aliphatic amines (**R‐NH_2_**) induces a thermodynamic amine exchange equilibrium with **MG‐DAE‐II_R_**, thus breaking the crosslinks. Blue irradiation of the equilibrium mixture kinetically traps the exchange product in the corresponding inactive aniline form, i.e., **MG‐DAE‐I_R_**. Ultimately, this approach provides photocontrol over crosslinking density and covalent binding/release of functional molecules to/from the microgel.

Herein, we present a novel photoswitchable DAE crosslinker for PVCL‐based microgels resembling the static aniline state of our prototypical “photoumpolung” system^[^ ^50^, ^51^ ^]^ (Figure 1). Ring‐closure of this core‐enriched motif (**MG‐DAE‐I_x_**) is induced by UV light irradiation, leading to the formation of ring‐closed imine **MG‐DAE‐II_x_**. Treatment of this now electrophilic species with primary aliphatic amines (**R‐NH_2_**) establishes a dynamic amine exchange equilibrium with **MG‐DAE‐II_R_**. Finally, blue light irradiation “freezes” the dynamic equilibrium and thus kinetically traps the exchange product in an inactive aniline state, that is, **MG‐DAE‐I_R_**. This approach enables photocontrol over the internal responsivity and the covalent binding of functional molecules to the microgel. In order to allow for a copolymerization with *N*‐vinylcaprolactam (**VCL**) and *N,N’‐*methylenebis(acrylamide) (**BIS**) from an aqueous medium, triethylene glycol moieties were attached to the DAE exterior as linker motifs between the photoactive core structure and two polymerizable methacrylamide units. Intrigued by related literature‐known examples showcasing multi‐responsive behavior of photoswitchable microgels with increasing photoswitch content,^[^ ^21^, ^22^, ^24^ ^]^ we targeted three microgels with DAE contents of 1, 2, and 3 mol% and investigated their stimuli‐responsiveness towards temperature and the pH value in the native as well as UV light activated state by means of UV/vis spectroscopy, dynamic light scattering (DLS), measurements of the electrophoretic mobility, as well as bright‐field scanning transmission electron microscopy (BFSTEM). To keep the overall crosslinker content of 3 mol% and thus the crosslinking density of these microgels constant, **BIS** was added to the polymerization mixture as well. In addition, one microgel sample containing the highest amount of photoswitchable DAE crosslinkers (**MG‐DAE‐I_x_ 3 mol%**) was synthesized without **BIS** to explore the light‐activated disassembly by primary amines. The potential of our microgels for drug delivery purposes was investigated by light‐gated exchange experiments with rather hydrophobic *n*‐octylamine (**Oct‐NH_2_**), hydrophilic 2‐(2‐(2‐(2‐aminoethoxy)ethoxy)ethoxy)ethan‐1‐ol (**TEG‐NH_2_**), and fluorinated 3,5‐bis(trifluoromethyl)benzyl amine (**BF‐NH_2_**) under mild (neutral) conditions in methanol. The exchange protocol utilized was optimized beforehand for a series of different aliphatic and aromatic amines using native **DAE‐I_x_**. Our method demonstrates a promising approach for the light‐controlled uptake of amines and illustrates the tremendous potential of photoswitchable microgels in materials and life science.

## Results and Discussion

### Light‐Gated Dynamic Covalent Amine Exchange

**Figure 2:**
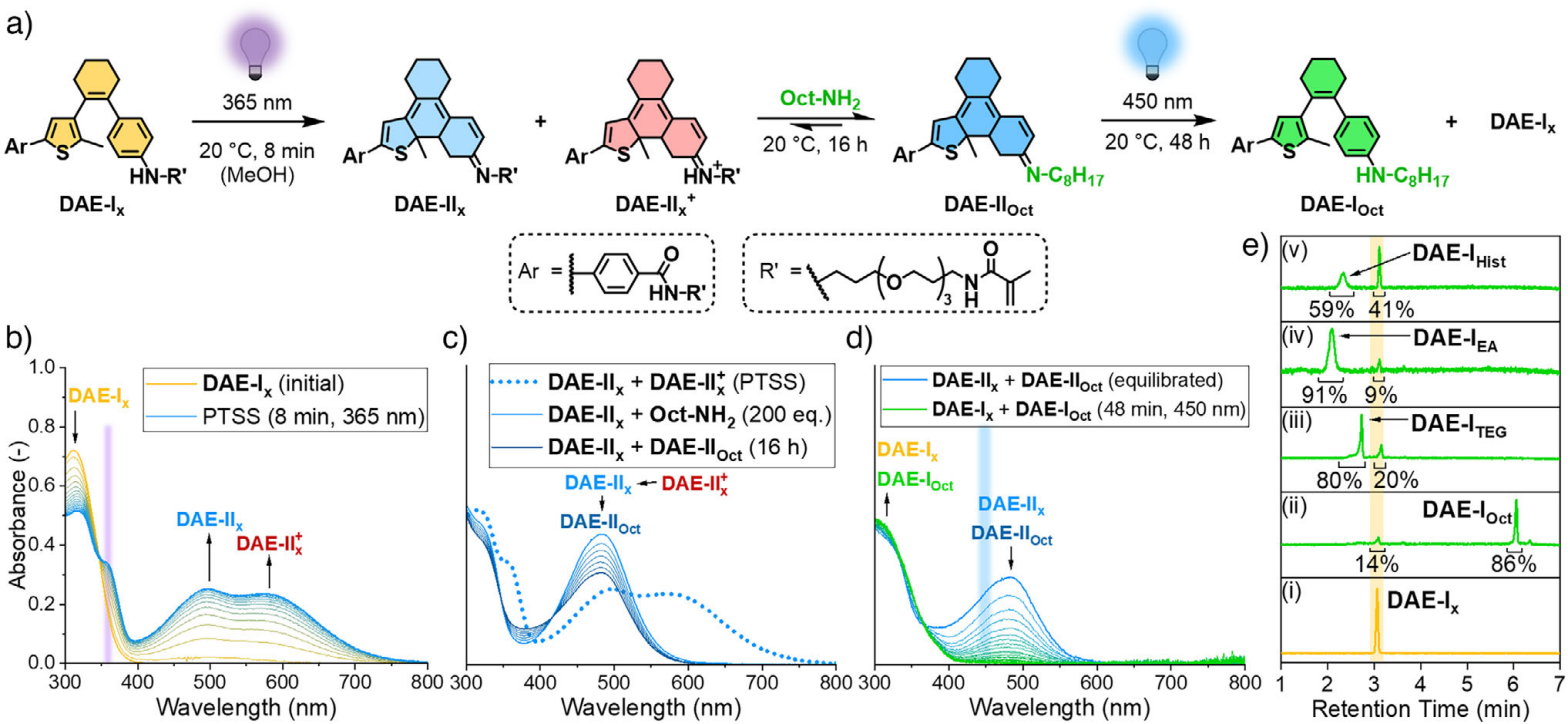
a) Schematic representation of light‐gated amine exchange reaction with crosslinker **DAE‐I_x_** as model compound for our PVCL‐based microgels. b) UV/vis absorption spectra of the 365 nm UV light induced ring‐closure of **DAE‐I_x_** (30 µM) in neutral methanol leading to the formation of imine **DAE‐II_x_** (480 nm) and iminium ion **DAE‐II_x_^+^** (585 nm) with near‐quantitative conversion of 98% in the PTSS. c) UV/vis absorption spectra of the 16 h thermal equilibration after addition of **Oct‐NH_2_** (200 eq.). d) UV/vis absorption spectra of the 450 nm blue light induced ring‐opening of the equilibrium composition between **DAE‐II_x_** and **DAE‐II_Oct_** leading to formation of **DAE‐I_x_** and **DAE‐I_Oct_**. e) UPLC traces of (i) **DAE‐I_x_** and the different amine exchange reactions investigated after blue light trapping containing (ii) 14% **DAE‐I_x_** and 86% **DAE‐I_Oct_** (for the exchange reaction with **Oct‐NH_2_**), (iii) 20% **DAE‐I_x_** and 80% **DAE‐I_TEG_** (for the exchange reaction with **TEG‐NH_2_**), (iv) 9% **DAE‐I_x_** and 91% **DAE‐I_EA_** (for the exchange reaction with ethylene diamine), and (v) 41% **DAE‐I_x_** and 59% **DAE‐I_Hist_** (for the exchange reaction with histamine).

To find optimal conditions for an exchange reaction to occur under mild conditions, model studies using native DAE crosslinker **DAE‐I_x_** were performed in the first place. According to Di Stefano and co‐workers,^[^ ^46^, ^47^ ^]^ this reaction can principally proceed for amines with similar p*K*_a_ values via a concerted proton transfer in an aminal intermediate formed from charge‐neutral imines without the requirement for acid catalysis. Therefore, methanol was chosen as a neutral and polar protic solvent. As a first step, the UV light induced 6π‐electrocyclization of **DAE‐I_x_** was investigated by irradiating a 30 µM solution at 365 nm (Figure 2a, b), displaying the formation of two new absorption bands in the visible region with maxima at 480  and 585 nm. These bands can be assigned to the formation of ring‐closed imine **DAE‐II_x_** and iminium ion **DAE‐II_x_^+^**. In order to prove this assumption, the same experiment was repeated in the presence of 5.0 vol% aqueous pH 4.0 and 10 buffer, which led to the formation of only **DAE‐II_x_^+^** in acidic and **DAE‐II_x_** in basic environment due to the forced protonation and deprotonation of the Schiff base, respectively (Figure S1C–D, Supporting Information). Overall, a conversion of up to 98% in the photothermal stationary state (PTSS) was achieved under neutral and acidic conditions according to ^1^H NMR spectroscopic and ultra‐high performance liquid chromatographic (UPLC) measurements (Figure S1A–B, Supporting Information). After reaching the PTSS, **Oct‐NH_2_** (200 eq.) was added causing an immediate hypsochromic shift of the 585 nm iminium band to the 480 nm imine band as a consequence of rapid deprotonation by the basic amine (Figure 2c). The imine band further decreased within a total of 16 h equilibration at 20 °C, which might be a result of the anticipated amine exchange to **DAE‐II_Oct_**. The formation of a corresponding iminium form of **DAE‐II_Oct_** similar to **DAE‐II_x_^+^**, however, cannot be observed under the experimental conditions due to the 200‐fold excess of basic **Oct‐NH_2_**. As the two imine forms, that is, **DAE‐II_x_** and **DAE‐II_Oct_**, exhibit almost identical absorption spectra in accordance to previous findings,^[^ ^50^ ^]^ a clear assignment to either one of the two species, however, is impossible. Furthermore, as they are in constant dynamic exchange, a direct analysis of the equilibrium composition by UPLC measurements is not feasible either, as this usually contains large amounts of water and acid in the eluent mixture. Instead, the equilibrated reaction mixture was irradiated with 450 nm blue light to induce 6π‐cycloreversion converting both imine isomers to their corresponding inactive aniline forms, that is, **DAE‐I_x_** and **DAE‐I_Oct_**, and thus to lock the composition of the dynamic equilibrium (Figure 2d). Analyzing this deactivated mixture by UPLC undoubtedly shows the occurrence of both of these species with an overall conversion of 86% to **DAE‐I_Oct_**, clearly confirming successful amine exchange (Figure 2e). The same protocol was further applied to **TEG‐NH_2_** and other primary aliphatic amines, that is, ethylene diamine and histamine, giving almost identical results with the highest conversion found for ethylene diamine (91%) and the lowest for histamine (59%) (Figure 2e). The first can easily be explained by the second amine group of ethylene diamine leading to a doubled effective concentration and the latter is most likely a result of a lower p*K*_a_ value and thus a reduced amine exchange reactivity of histamine's primary amine group. Furthermore, this specific reaction was equilibrated for only 1 h at 20 °C since no further spectral changes were observed after this time. Besides the discussed aliphatic amines, aromatic indoline and 5‐trifluoromethylindoline were also tested for a potential amine exchange reaction with **DAE‐II_x_** yet without success (Table S1, Supporting Information), as a result of their even lower p*K*_a_ values and thus insufficient reactivity compared to the aliphatic amine leaving group of **DAE‐II_x_**, which is known in the literature.^[^ ^52^ ^]^ Furthermore, the impact of Brønsted acid catalysis on the exchange reaction was investigated by adding either 5.0 vol% of an aqueous pH 4.0, 5.0, or 7.0 buffer or 0.1 vol% organic acetic (AcOH) or trifluoroacetic acid (TFA) to the PTSS mixture prior to treatment with the corresponding amine (Table S1, Supporting Information). However, amine exchange was not observed in either of these cases, most likely pointing at a deactivation of the attacking amine by protonation to the corresponding ammonium salt.

### Synthesis and Stimuli‐Responsive Behavior of DAE‐Crosslinked Microgels

**Figure 3:**
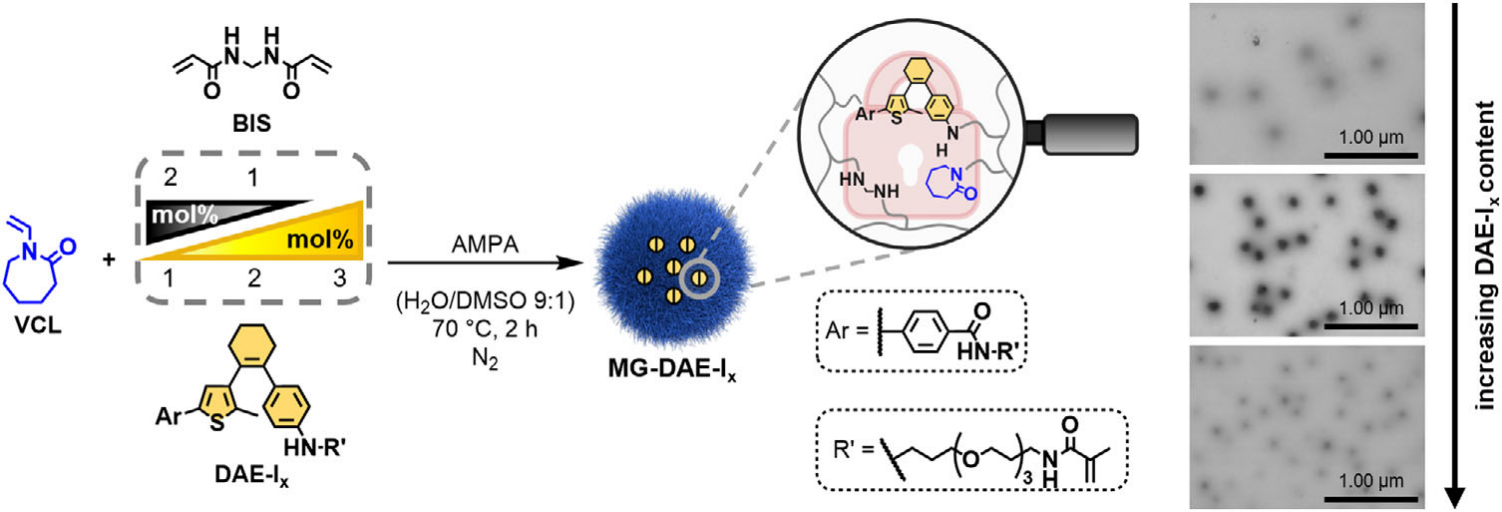
Synthesis of microgels containing crosslinker **DAE‐I_x_** via batch precipitation polymerization initiated by 2,2′‐azobis(2‐amidinopropane) dihydrochloride (AMPA). To keep a total of 3 mol% crosslinks constant, different ratios of **BIS** and **DAE‐I_x_** (2:1, 1:2, and 0:3 mol%/mol%) were used.

The light‐gated exchange of our DAE crosslinker with various primary aliphatic amines in high conversion clearly demonstrates the potential of **DAE‐I_x_** to be employed as photodynamic crosslinker and binding site in microgels. Thus, three PVCL‐based microgels (**MG‐DAE‐I_x_**) with 1, 2, and 3 mol% of synthesized crosslinker **DAE‐I_x_** were prepared via batch precipitation polymerization in aqueous solution. Since all microgels should have a constant crosslinker content of 3 mol%, a differential amount of non‐degradable crosslinker **BIS** was added to the microgels with lower **DAE‐I_x_** content (Figure 3). The polymerization was initiated with 2,2′‐azobis(2‐methylpropionamidine) dihydrochloride (AMPA) and DMSO (10 vol%) was used to ensure sufficient solubility of **DAE‐I_x_** during the reaction. In batch precipitation polymerization, the localization of **DAE‐I_x_** in the microgel core results from its bivalent structure, which leads to higher segment densities in the interior since the methacrylamide groups react faster than the vinyl groups of VCL.^[^ ^53^, ^54^ ^]^ In addition, the hydrophobic character of **DAE‐I_x_** promotes the accumulation in the microgel core. Moreover, a pure PVCL microgel, that is, **MG‐PVCL**, containing 3 mol% **BIS** without **DAE‐I_x_** was prepared and used as a reference for further investigations (Experimental Section, Supporting Information). Overall, the yields were found to decrease slightly with increasing photoswitch content compared to **MG‐PVCL** (Table S2, Supporting Information).

After successful synthesis of the microgels, the incorporated content of **DAE‐I_x_** was evaluated by means of UV/vis spectroscopy. This method was chosen as it provides exceptionally high sensitivity for the rather low content of the photochromic compound that is the only species in the microgels that absorbs light above 300 nm. Plus, its extinction coefficient can easily be calculated from a solution of native **DAE‐I_x_** in methanol at various concentrations (Figure S2A, Supporting Information). However, due to the scattering background of the microgels, it is impossible to measure the absorbance of the DAE in the microgels directly. Instead, the measured extinction of the photoactive microgels were corrected for the PVCL scattering to extract the pure absorbance of **DAE‐I_x_** within the according samples. To this end, the scattering curve of reference **MG‐PVCL** was measured at different concentrations in the range between 50 and 1050 µg mL^−1^ (Figure S2B, Supporting Information). Here, a linear correlation was confirmed, which is the basic requirement for the spectral correction. With this information, the specific scattering pattern of each DAE‐crosslinked microgel was calculated and subtracted from the measured extinction (Figure S2C–E, Supporting Information). This way, the pure absorbance of **DAE‐I_x_** was obtained and thus its content in the microgels could be calculated using the determined extinction coefficient of 20 900 ± 900 L mol^−1^ cm^−1^ at 314 nm (Table S3, Supporting Information). In summary, a successful and highly efficient incorporation of 85% to ∼100% of the targeted 1, 2, and 3 mol% of DAE incorporation was achieved with absolute values of 0.85, 2.08, and 2.42 mol%, highlighting the advantage of a direct one‐step copolymerization, especially with regard to the quantity of photoswitchable compound used, over an alternative two‐step post‐functionalization route that heavily relies on the efficiency of both individual steps.^[^ ^21^, ^22^, ^24^ ^]^ For simplicity, the microgels are referred to as **MG‐DAE‐I_x_ 1 mol%**, **MG‐DAE‐I_x_ 2 mol%**, and **MG‐DAE‐I_x_ 3 mol%** in the following.

**Figure 4:**
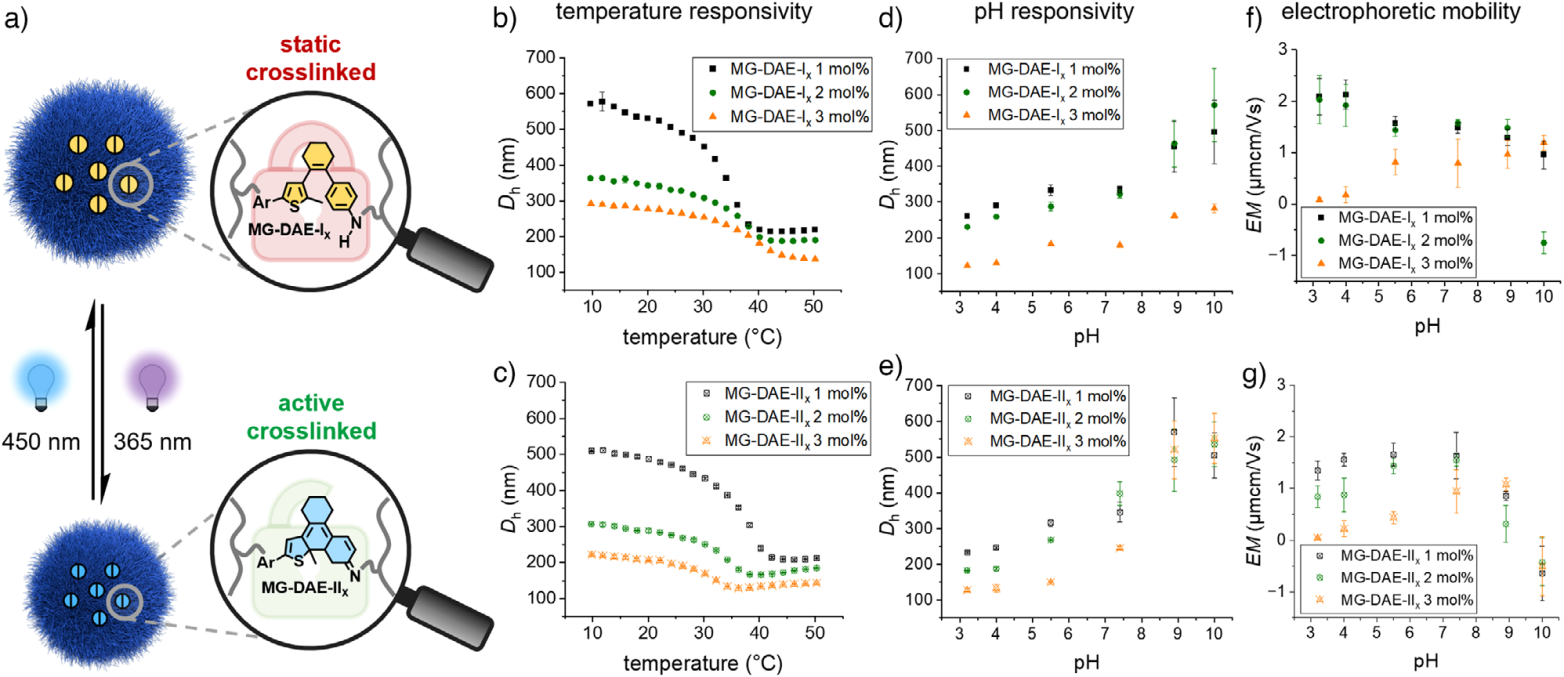
a) Reversible photoswitching of **DAE‐I_x_**‐crosslinked microgels from the static ring‐open aniline (top) to the dynamic ring‐closed imine state (bottom). On the top, the heating curve of the temperature‐responsive behavior (b) in HPLC water from 10 °C to 50 °C as well as the pH‐responsivity (d) of the hydrodynamic diameter (*D*_h_) and the electrophoretic mobility (*EM*) (f) of the **MG‐DAE‐I_x_** microgels in the static ring‐open state at different pH values (3.2–10.0) in buffered solutions at 50 °C are shown, whereas at the bottom, the respective responsivities (c, e, and g) of the switched **MG‐DAE‐II_x_** microgels after irradiation with 365 nm UV light are displayed.

Subsequently, BFSTEM images were recorded to investigate the microgel formation, particle size, and the influence of the DAE content on the morphology of the synthesized microgels (Figure S3A‐C, Supporting Information). Here, a narrow size distribution is observed for all **MG‐DAE‐I_x_** microgels, and the particle size decreases with increasing DAE content from 272.0 ± 26.4, 155.7 ± 14.9 to 112 ± 12.3 nm (Table S4, Supporting Information). Thus, the BFSTEM images confirm the formation of spherical microgels in the dry state. For further evaluation of the size and swelling capacity, the microgels were investigated with regard to their temperature, pH, and ionic strength responsivity at different switching states of the DAE crosslinker in order to determine the influence of photoisomerization (Figure 4). For this purpose, the hydrodynamic diameters (*D*_h_) were determined in heating and cooling curves at temperatures between 10 °C and 50 °C (Figure 4b,c) in water and at pH values between 3.2 and 10.0 (Figure 4d,e) using DLS.

Since PVCL polymers have a lower critical solution temperature (LCST) at 32 °C,^[^ ^55^, ^56^ ^]^ the microgels are therefore expected to show a VPTT with LCST‐like behavior around this temperature, being in a swollen state below and in a collapsed state above the VPTT. The *D*_h_ values of the heating curves of the **MG‐DAE‐I_x_** microgels showed temperature‐responsive behavior depending on the DAE content: The lower the content, the greater the swelling behavior and the further the shift of the VPTT towards lower temperatures of 37.5 °C, 33.6 °C, to 32.9 °C (for DAE contents of 3, 2, and 1 mol%, Figure 4b). Thus, at 20 °C, the *D*_h_ values are between 280 and 530 nm and at 50 °C only between 140 and 220 nm. In this context, the *D*_h_ values of the collapsed microgels are in accordance with the measured particle diameters in the dry state from the BFSTEM images (cf. Figure S3, Supporting Information). When the microgels were irradiated with 365 nm UV light, as in the previous experiments, this causes ring‐closure of bound **DAE‐I_x_** to **DAE‐II_x_** and – under neutral and acidic conditions (pH ≤ 7.4) – **DAE‐II_x_^+^** (cf. Figures 2a,b  and S7, Supporting Information). As a result, the microgels shrink displaying *D*_h_ values between 210 and 490 nm at 20 °C (Figure 4c). Furthermore, the swelling behavior in the heating curves again decreases with increasing DAE content, and above the VPTT at 50 °C the *D*_h_ values become similar to the non‐irradiated microgels. Nevertheless, ring‐closure leads to a VPTT shift to lower temperatures of 34.7 °C, 31.7 °C to 29.6 °C that is more pronounced with increasing DAE content (1, 2 to 3 mol%), contrary to the ring‐open state. This can be explained by stronger hydrophobic interactions of the fully π‐conjugated planar ring‐closed isomer, causing the change in VPTT.^[^ ^23^, ^57^ ^]^ In addition, the cooling curves of the microgels in the ring‐open and ring‐closed DAE states show a nearly congruent trend to the heating curves, thus the temperature‐responsive behavior of the microgels is completely reversible (cf. Figure S4, Supporting Information). Next, the influence of the ionic strength on the *D*_h_ of the **MG‐DAE‐I_x_** samples at 20 °C was investigated, revealing a minor shrinkage of the microgels even at low concentrations of 10 mM (Figure S5A, Supporting Information). In order to minimize the influence of ionic strength, buffer solutions with an accordingly low ionic strength of 10 mM were prepared (Experimental Section, Supporting Information). Due to the localization of the photoswitch in the microgels, the biggest impact of the pH is assumed to occur in the microgel core. To evaluate the responsivity in the core, the *D*_h_ values were measured at different pH values at 50 °C where the microgels are in their collapsed state (Figure 4d,e). In the ring‐open DAE state (i.e., **MG‐DAE‐I_x_**), the *D*_h_ values in acidic buffer increase slightly towards neutral pH values and with further increasing basicity the *D*_h_ increase becomes even stronger, while the different microgels with 1, 2, and 3 mol% **DAE‐I_x_** can still be clearly distinguished (Figure 4d). In the ring‐closed state, the microgels are overall slightly smaller, as mentioned before, but show a similar trend in pH value dependency, although in basic buffers, all **MG‐DAE‐I_x_** microgels exhibit similar *D*_h_ values (Figure 4e). In both switching states of the DAE crosslinker, the polydispersity index (PDI) of the microgels remained small at all pH values, indicating a narrow size distribution (Tables S5, S6, Supporting Information). Furthermore, measurements at temperatures below the VPTT showed no effect of the pH on the *D*_h_ regardless of the switching state, thus indicating that pH responsivity indeed occurs only in the microgel core (Figure S5B,C, Supporting Information). To gain a better understanding of this complex behavior, electrophoretic light scattering (ELS) was performed at the pH values investigated before to determine the effective surface charge of the microgels. Since a charge should only be caused by the photoswitchable motif in the core, we expected the microgels to have no surface charge at temperatures below the VPTT, which was confirmed by the respective ELS measurements (Figure S5D, Supporting Information). Therefore, ELS measurements were carried out in the collapsed state of the microgels to approximate the surface charge (Figure 4f,g). Here, an electrophoretic mobility (*EM*) of ≈ 2.0 µm cm Vs^−1^ was measured for **MG‐DEA‐I_x_ 1 mol%** and **MG‐DEA‐I_x_ 2 mol%**, which decreases slightly with increasing pH values but remains positive overall (Figure 4f). Only **MG‐DEA‐I_x_ 3 mol%** shows low mobility at acidic pH values, which increases and becomes positive with increasing pH values until finally approaching the *EM* (≈ 1.0 µm cm Vs^−1^) of the other microgels. After UV light irradiation of the microgels, a half‐pronounced *EM* was measured for **MG‐DEA‐II_x_ 1 mol%** and **MG‐DEA‐II_x_ 2 mol%** at acidic pH values. Furthermore, the *EM* increased until pH 7.4 and then declined to a negative *EM* of ≈ ‐1.0 µm cm Vs^−1^ for these two microgels at basic pH values (Figure 4g). As before, a neutral *EM* was initially measured for **MG‐DEA‐II_x_ 3 mol%** at pH 3.2, but then the *EM* also increased directly until pH 7.4 and at basic pH values it also became negative (≈ ‐1.0 µm cm Vs^−1^). In general, the results indicate that charges are located in the microgel core in alignment with previous findings. The repulsive electrostatic interactions between the same charged species and the resulting osmotic pressure of the native counterions lead, on the one hand, to a swelling of the microgels.^[^ ^58^ ^]^ On the other hand, the measurements are carried out at temperatures above VPTT, which causes the microgels to collapse. These two simultaneously occurring effects interfere with each other and can thereby cause the partial deviations within the DLS and ELS measurements as well as the slightly contradictory results. Moreover, UV light irradiation generates ring‐closed Schiff base **MG‐DAE‐II_x_**, which potentially accumulates net positive charges in the microgel core in an acidic environment (Figure 4d–g). To prove this hypothesis, ring‐closure of **MG‐DAE‐I_x_** was additionally followed by UV/vis spectroscopy under aqueous buffered conditions to confirm the previously described behavior. Indeed, formation of solely iminium ion **MG‐DAE‐II_x_^+^** at acidic pH values (pH 3.2, 4.0, and 5.5), the formation of both imine **MG‐DAE‐II_x_** and iminium **MG‐DAE‐II_x_^+^** under neutral conditions (pH 7.4 and HPLC water), and the formation of purely imine **MG‐DAE‐II_x_** at basic pH values (pH 8.9 and 10) could be followed nicely by monitoring the corresponding absorbance bands at 480 nm (**MG‐DAE‐II_x_**) and 585 nm (**MG‐DAE‐II_x_^+^**) in the visible region (Figure S7, Supporting Information). Furthermore, an overall reduced intensity of the visible bands and a broad absorbance around 400 nm in neutral and basic aqueous environments was noted. This fatigue can be explained either by a UV light induced ring annulation of the ring‐closed imine isomer that is widely known in the literature^[^ ^59^ ^]^ (cf. Figure S1C‐E, Supporting Information), or its potential rearrangement to a thioenolate motif,^[^ ^60^, ^61^ ^]^ which could further explain the occurrence of net negative charge and thus the *EM* of ≈ ‐1.0 µm cm Vs^−1^ at basic pH values. Moreover, due to the limited penetration depth of UV light in the microgel, the maximum conversion in the PTSS might be reduced as well. Either way, indicated by the considerably low absorbance of the ring‐closed **MG‐DAE‐II_x_** microgel states at basic pH values, a degradation of the DAE unit in the microgel environment according to one of these mechanisms leading to a loss of photoactive material cannot be ruled out at this point. Nevertheless, these results clearly demonstrate that switching of the DAE crosslinker allows for a remotely controlled internal pH and temperature responsivity of our microgels.

### Light‐Gated Post‐Modification of DAE‐Crosslinked Microgels

**Figure 5:**
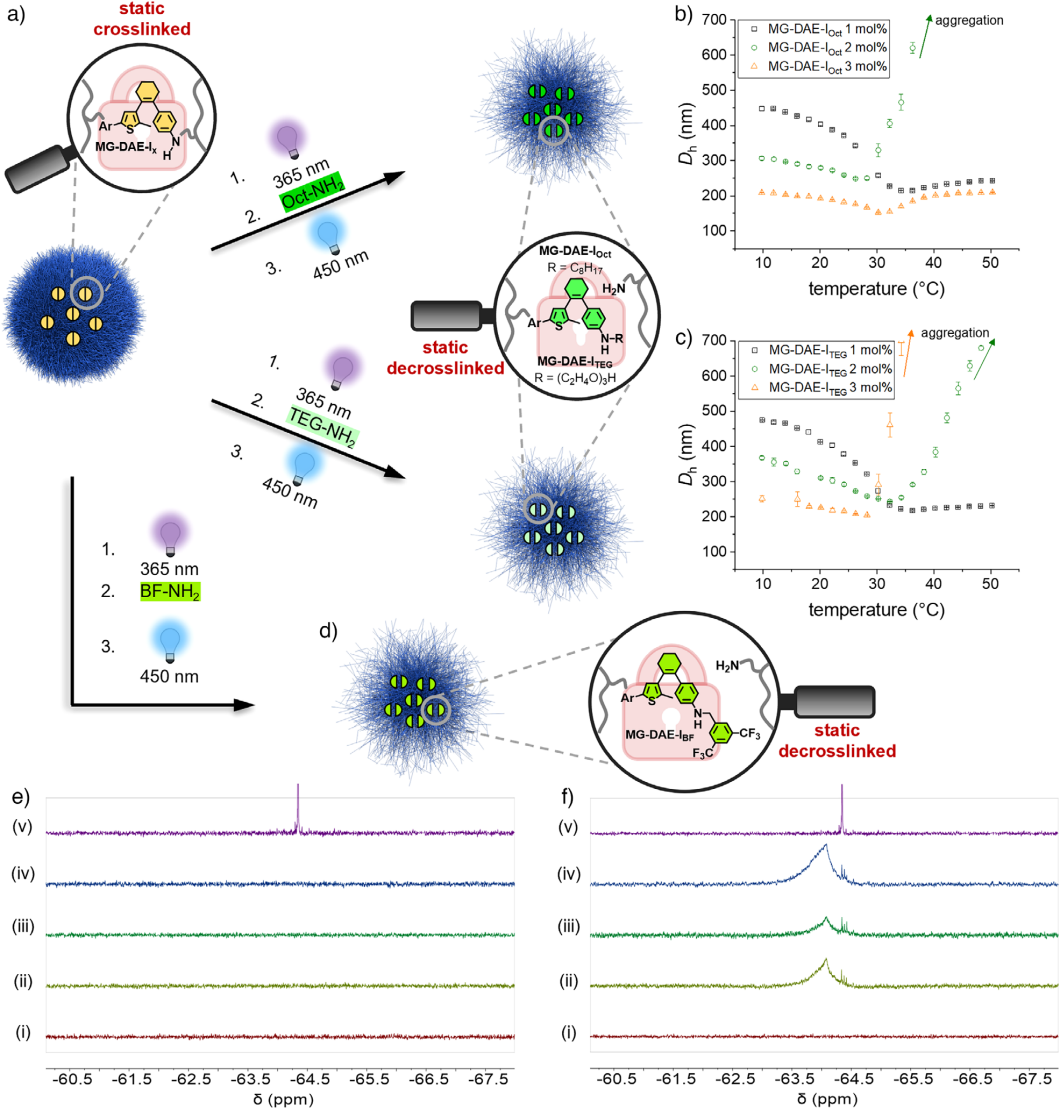
a) Breaking inner microgel crosslinks by light‐gated amine exchange with hydrophobic **Oct‐NH_2_** or hydrophilic **TEG‐NH_2_**. Influence of incorporated hydrophobic (b) and hydrophilic (c) alkyl chains on the temperature‐responsivity of the heating curves of the locked and inactive **MG‐DAE‐I_Oct_** and **MG‐DAE‐I_TEG_** microgels. d) Confirming the covalent binding of functional **BF‐NH_2_** in the microgels after light‐gated exchange using ^19^F NMR. The ^19^F{^1^H} NMR spectra of (i) **MG‐PVCL** as well as the three DAE‐containing microgels with (ii) 1 mol%, (iii) 2 mol%, and (iv) 3 mol% as well as (v) **BF‐NH_2_** before (e) and after (f) light‐gated amine exchange are shown.

Intrigued by the multi‐responsive behavior of our microgels and the apparently high thermal stability of the upon UV light activation formed *C═N* bond towards hydrolysis (cf. Figure S7, Supporting Information), their potential for light‐gated reversible covalent binding of functional primary amines, for example, for drug delivery purposes was further investigated. To this end, the optimized protocol for amine exchange with primary **Oct‐NH_2_** and **TEG‐NH_2_** as representatives of rather hydrophobic and hydrophilic building blocks was applied to our three **MG‐DAE‐I_x_** microgels. Therefore, the microgels were redispersed in methanol and irradiated with 365 nm followed by the addition of the respective amines in excess (cf. Experimental Section and Figure S8, Supporting Information). Afterwards, we irradiated the “loaded” microgels with 450 nm to bring the dynamic equilibrium to a hold and lock the photoswitchable exchange product in its inactive ring‐open state, that is, **MG‐DAE‐I_Oct_** and **MG‐DAE‐I_TEG_** (Figure 5a). Moreover, methanol as solvent has the beneficial advantage to cause a higher degree of swelling than water, whereby no temperature responsivity of the microgels occurs in this solvent (Tables S7, S8, Supporting Information).^[^ ^4^, ^62^ ^]^ The increased swelling leads to a higher translucency, thus enhancing light penetration, which in turn significantly reduces light scattering of the microgel network, making practically all the light available for switching the incorporated DAE crosslinker similar to switching pure **DAE‐I_x_**.^[^ ^25^ ^]^ This effect ensures high photoconversions in the microgel environment.

After light‐gated amine exchange, we first investigated the influence of the DAE crosslinker breaking on the morphology of the microgels using BFSTEM (Figure S3D–I, Supporting Information). This revealed that all microgels maintained their spherical shape and kept a narrow size distribution. The particle size in the dried state of the microgels is between 120 and 220 nm regardless of the amines used for the exchange reaction (Table S4, Supporting Information). From these results, we can conclude that not all covalent crosslinks of the photoswitch were broken, which can again be explained by an incomplete ring‐closure and thus activation in the PTSS or photodegradation.^[^ ^59^, ^60^, ^61^ ^]^ This is additionally supported by a substantially lower absorbance in the visible region of all microgels and in particular the samples containing 1 mol% and 2 mol% DAE crosslinker (Figure S8B–D) in comparison to our model studies (cf. Figure 2c,d), which indicates a lower concentration of active imine species. Furthermore, additional physical crosslinks could be established due to the formation of hydrogen bonds by free amine groups resulting from decrosslinking and the hydrophobic interactions induced by incorporated alkyl chains in the microgel network. Otherwise, especially in the case of **MG‐DAE‐I_x_ 3 mol%** where only **DAE‐I_x_** was used as a crosslinker, degradation of the microgels would typically be expected. Subsequently, the influence of the hydrophobic and hydrophilic amines on the temperature‐responsive behavior of the microgels was examined at temperatures between 10 °C and 50 °C in HPLC water via DLS (Figure 5b,c). Here, the heating curves of the **MG‐DAE‐I_Oct_** microgels, in which the amine was exchanged with **Oct‐NH_2_**, exhibit *D*_h_ values between 200 and 450 nm at temperatures below the VPTT in the swollen state, whereby the dependence on the DAE content remains unchanged (Figure 5b). With increasing temperatures, the microgels begin to collapse and the *D*_h_ values decrease. However, as soon as the VPTT is exceeded, the *D*_h_ values for **MG‐DAE‐I_Oct_** **1 mol%** and **MG‐DAE‐I_Oct_ 3 mol%** increase again a little due to slight agglomeration. Additionally, the corresponding cooling curves show complete reversibility of this process (Figure S6A, Supporting Information). In contrast, strong aggregation occurs for **MG‐DAE‐I_Oct_ 2 mol%** as demonstrated by the irreversible nature of the respective cooling curve (Figure S6A, Supporting Information). For the **MG‐DAE‐I_TEG_** microgels, the *D*_h_ values of the heating curves in the swollen state are between 250 and 480 nm (Figure 5c). In this case, **MG‐DAE‐I_TEG_** **2 mol%** and **MG‐DAE‐I_TEG_ 3 mol%** are observed to aggregate when the VPTT is exceeded, which again can be seen by the remaining or even increasing aggregation in the cooling curves (Figure S6B, Supporting Information). Only **MG‐DAE‐I_TEG_** **1 mol%** does not show this behavior but displays a constant *D*_h_ around 230 nm above the VPTT, which is reversible according to the respective cooling curve (Figure S6B, Supporting Information). When comparing the **MG‐DAE‐I_Oct_** and **MG‐DAE‐I_TEG_** microgels with the respective DAE contents in the swollen state, it can be ascertained that the *D*_h_ values are 25 to 60 nm larger for the **MG‐DAE‐I_TEG_** microgels at 10 °C. The stronger swelling of the **MG‐DAE‐I_TEG_** microgels most likely results from the hydrophilic **TEG‐NH_2_** that is able to form additional hydrogen bonds with water molecules and the polymer network of the microgel. In contrast, the **MG‐DAE‐I_Oct_** microgels exhibit a lower degree of hydrogen bonding due to the hydrophobic **Oct‐NH_2_** and hence show less swelling. Moreover, the swelling behavior of the microgels after amine exchange in methanol at 20 °C and 50 °C was investigated (Tables S9, S10, Supporting Information). As expected, the microgels showed no temperature responsiveness and no aggregation was observed. Furthermore, the increased swelling of the **MG‐DAE‐I_TEG_** compared to the **MG‐DAE‐I_Oct_** microgels was verified in methanol. These results demonstrate that even small amounts (1–3 mol%) of the DAE crosslinker are sufficient to influence the swelling capacity of microgels in the core region by light‐gated amine exchange of hydrophobic and hydrophilic amines.

Successful incorporation of the amines was in addition indicated by UV/vis spectra measured for the respective switching states of the DAE‐crosslinked microgels occurring during light‐gated amine exchange. In general, irradiation of a 0.5–1.0 mg mL^−1^ methanol solution of the corresponding microgel with 365 nm UV light caused formation of both imine **MG‐DAE‐II_x_** and iminium ion **MG‐DAE‐II_x_^+^** (Figure S8A, Supporting Information). The addition of the respective amine and thermal equilibration for 16 h at 20 °C led to an instantaneous blue shift of the 585 nm iminium band to the 480 nm imine band (Figure S8B,C, Supporting Information). Additionally, a slight decrease of the latter band over the course of the equilibration was observed, thus indicating successful amine exchange to **MG‐DAE‐II_Oct_** and **MG‐DAE‐II_TEG_**. In contrast to the experiments with native **DAE‐I_x_**, 450 nm blue light irradiation led to the expected decrease of the 480 nm band and the increase of the 314 nm band of **MG‐DAE‐I_Oct_** and **MG‐DAE‐I_TEG_**, but with a significantly lower overall intensity of the latter (Figure S8B,C, Supporting Information). Again, this is most likely a result of either photodegradation,^[^ ^59^ ^]^ thioenolate formation,^[^ ^60^, ^61^ ^]^ or limited conversion in the PTSS within the microgel network. In order to clearly assign the effects of this light‐gated process on the microgel properties to the anticipated amine exchange, that is, the associated reduction of covalent crosslinks and incorporation of the functional amine, **MG‐PVCL** was treated with **Oct‐NH_2_** yet leading to no obvious changes in the scattering pattern (Figure S9, Supporting Information) and the size of this microgel (Table S11, Supporting Information). Consequently, the observed effects can be unambiguously attributed to the proposed exchange.

Since the UV/vis spectra do not provide direct evidence for covalent incorporation of the specific amines into the microgel network, we sought to undoubtedly confirm their light‐gated incorporation. For this reason, the same amine exchange procedure was applied for **BF‐NH_2_** as this introduces six fluorine atoms to the microgels that can easily be identified by ^19^F NMR spectroscopy (Figure 5d). On the first glance, this process revealed the same trends in the UV/vis measurements as for **Oct‐NH_2_** and **TEG‐NH_2_** (Figure S8D, Supporting Information), again indicating the universal incorporation of amines into our developed system. Next, the ^19^F NMR investigation of the three DAE‐crosslinked microgels and **MG‐PVCL** before and after the overall process revealed successful incorporation of **BF‐NH_2_** into the photoactive samples, as nicely shown by a broad polymeric signal between ‐63.5 and ‐64.5 ppm found only in the DAE‐containing samples (Figure 5e,f). Thus, equally successful incorporation of **Oct‐NH_2_** and **TEG‐NH_2_** and therefore a reduction of the covalent crosslinks in the microgels can be assumed as well. Furthermore, the locked **MG‐DAE‐I_BF_** microgels were exposed to a second amine exchange cycle using **Oct‐NH_2_** to trigger **BF‐NH_2_** release. For this purpose, the dialyzed samples were redispersed in methanol, treated according to the general procedure for light‐gated **BF‐NH_2_** release (Experimental Section, Supporting Information), and submitted for ^19^F NMR analysis (Figure S10, Supporting Information). As clearly indicated by the intensity reduction of the polymeric signal of all three samples, successful but yet not quantitative release of **BF‐NH_2_** was achieved, which is most likely again caused by fatigue or an insufficient activation due to a shorter UV irradiation time (60 s instead of 90–120 s) compared to previous experiments. Nevertheless, these results unambiguously highlight the potential of our DAE‐crosslinked microgel system to take up and release any functional amine in a reversible and light‐controlled fashion.

## Conclusion

In this work, we establish a novel method for light‐gated reversible exchange of aniline‐type **DAE‐I_x_** with primary aliphatic amines under mild conditions based on our previous “photoumpolung” design.^[^ ^50^, ^51^ ^]^ It was shown by UV/vis, NMR, and UPLC investigations that UV light induced ring‐closure leads to the formation of electrophilic imine **DAE‐II_x_**, which after treatment with functional amines and blue light induced ring‐opening of the DAE yields the exchange products in their inactive aniline forms. By decorating **DAE‐I_x_** with two methacrylamide units on both sides of the photoactivatable *C‐N* bond, easy and highly efficient (85%–100% incorporation of targeted 1, 2, and 3 mol%) copolymerization with **VCL** and secondary crosslinker **BIS** was achieved, yielding a series of three **MG‐DAE‐I_x_** microgels with DAE crosslinks located in the core. Studying their stimuli‐responsiveness by DLS and ELS before and after ring‐closure of the DAE motif revealed a shrinkage (7–25% in HPLC water at 20 °C) of the microgels upon photoisomerization and a shift of their VPTT to lower temperatures (∼2‐8 °C). Furthermore, a generally greater swelling of the activated microgels with increasing pH values was found accompanied by positive to negative effective surface charges in the core, which is attributed to the increasing deprotonation of the formed Schiff base. Applying our protocol for light‐gated amine exchange to the microgels using three model amines (**Oct‐NH_2_**, **TEG‐NH_2_**, and **BF‐NH_2_**) revealed a more pronounced swelling after exchange with hydrophilic **TEG‐NH_2_** compared to hydrophobic **Oct‐NH_2_**. Additionally, successful post‐modification of the microgels as well as amine release could be verified by ^19^F NMR spectroscopy for **BF‐NH_2_** exchange and thus light‐controlled breaking of the DAE crosslinks and the incorporation of the investigated amines in our microgels was confirmed. Our study clearly demonstrates the enormous potential of DAEs to remote‐control the multi‐responsivity of modular PVCL‐based microgels and their capability to uptake, covalently bind, and release functional building blocks by the interplay of light with a certain chemical stimulus, that is, the presence of primary amines. This highlights an important step towards the development of photoswitchable microgel systems for applications at the interface of photochemistry, materials, and life sciences.

## Supporting Information

The authors have cited additional references within the Supporting Information.^[^ ^50^, ^51^, ^60^, ^61^, ^63^, ^64^, ^65^, ^66^, ^67^, ^68^, ^69^, ^70^, ^71^, ^72^, ^73^ ^]^

## Author Contributions

K.B. and F.G. were contributing equally to this work by developing the concept and carrying out experiments. S.E. synthesized the photoswitchable crosslinker and analyzed its photoisomerization by ^1^H NMR spectroscopy. J.D. supported amine exchange optimization. A.P. and S.H. supervised the study. K.B. and F.G. wrote the manuscript. All authors discussed the results and reviewed the manuscript.

## Conflict of Interests

The authors declare no conflict of interest.

## Supporting information



Supporting Information

## Data Availability

The data that support the findings of this study are available from the corresponding author upon reasonable request.
