# Supplementary material for: Light‐Gated Amine Exchange in Diarylethene‐Crosslinked Microgels
Source: Angew Chem Int Ed Engl. 2025 Aug 22;64(39):e202510141. doi: 10.1002/anie.202510141 (PMC12455440; doi:10.1002/anie.202510141)
Supplement: Supplementary file 1 — Supporting Information [file ANIE-64-e202510141-s001.docx]

Light-Gated Amine Exchange in Diarylethene-Crosslinked Microgels

Kevin Broi,^+^ Frédéric Grabowski,^+^ Sarah Esser, Jannick Dörr, Andrij Pich,* and Stefan Hecht*

[^+^] These authors contributed equally to this work.

[^*^] Corresponding authors.

**Table of Contents**

[Experimental Section S2](#_Toc199966940)

[Materials S2](#_Toc199966941)

[General Procedure for Amine Exchange and Investigated Conditions S2](#_Toc199966942)

[Ultraviolet/visible (UV/vis) Spectroscopy S2](#_Toc199966943)

[Nuclear Magnetic Resonance (NMR) Spectroscopy S2](#_Toc199966944)

[Ultra-High Performance Liquid Chromatography (UPLC) S3](#_Toc199966945)

[Medium Performance Liquid Chromatography (MPLC) S3](#_Toc199966946)

[High-Resolution Mass Spectrometry (HR‑MS) S3](#_Toc199966947)

[Dynamic Light Scattering (DLS) S3](#_Toc199966948)

[Electrophoretic Light Scattering (ELS) S3](#_Toc199966949)

[Bright‑Field Scanning Transmission Electron Microscopy (BFSTEM) S3](#_Toc199966950)

[Synthesis of Diarylethene Crosslinker **DAE‑I_x_** S3](#_Toc199966951)

[Synthesis of Photoswitchable **MG‑DAE‑I_x_** Microgels S10](#_Toc199966952)

[General Procedure for Amine Exchange within DAE-Crosslinked Microgels S10](#_Toc199966953)

[General Procedure for Light-Gated **BF‑NH_2_** Release S10](#_Toc199966954)

[Photoisomerization of **DAE‑I_x_** and Fatigue Measurements S11](#_Toc199966955)

[Content Determination of **DAE‑I_x_** in the Synthesized Microgels S12](#_Toc199966956)

[BFSTEM Images of Photoswitchable Microgels S13](#_Toc199966957)

[Responsivity of DAE-Crosslinked Microgels S14](#_Toc199966958)

[Photoisomerization in Buffered Media S16](#_Toc199966959)

[Light-Gated Amine Exchange inside the DAE Microgels S17](#_Toc199966960)

[Blank Experiment with **MG‑PVCL** S17](#_Toc199966961)

[Light-Gated Release of **BF‑NH_2_** S18](#_Toc199966962)

[NMR Spectra S19](#_Toc199966963)

[References S31](#_Toc199966964)

Experimental Section

Materials

Chemicals were purchased from Sigma Aldrich, ChemSolute, VWR Chemicals, TCI Chemicals, Carl Roth, BLDpharm, Merck Millipore, Fisher Scientific, and ABCR Chemical and were used as received if not other stated. Deuterated solvents were purchased from Eurisotop or Deutero GmbH and Sigma Aldrich. Spectroscopy and HPLC grade solvents were purchased from Merck Millipore and VWR Chemicals. Technical grade solvents were distilled prior to use. *N*-vinylcaprolactam (**VCL**) was distilled and recrystallized from hexane before use. All buffer solutions were adjusted to an ionic strength of *I* = 10 mM. The buffer solutions from pH 3.2 – 10 were prepared according to the literature.^[1]^ The different buffer systems were limited to formic acid (pH 3.2 – 4.0), succinic acid (pH 5.5), phosphate (pH 7.4), tris(hydroxymethyl)aminomethane (TRIS, pH 8.9), and carbonate (pH 10). The different ionic strengths were adjusted with sodium chloride and HPLC grade water.

General Procedure for Amine Exchange and Investigated Conditions

Amine exchange reactions were optimized on a 30 µM scale as this mimics the operation conditions of our microgel studies closely. In general, **DAE‑I_x_** (559 µg, 0.60 µmol, 1.0 eq.) was dissolved in spectroscopy grade methanol (20 mL). After sonication for 3 min, either 0.1 vol% organic acid (AcOH or TFA) or 5.0 vol% aqueous buffer (pH 4.0 or pH 5.5) or no further ingredient were added to 3 mL of this solution and the mixture was irradiated with 365 nm UV light until no further spectral changes were observed. Afterwards, 200 eq. of the respective amine were added and the mixture was equilibrated for 1 – 16 h at 20 °C. The mixture was then irradiated with 450 nm blue light until no further spectral changes were observed. Afterwards, a 100 µL sample was submitted for UPLC analysis.

Table S1. Investigated conditions for amine exchange model studies with DAE‑I_x_.

| Ingredient | Amine | Result^[a]^ |
| --- | --- | --- |
| pH 4.0 buffer (5.0 vol%) | *n*‑propylamine, indoline | very slow hydrolysis of **DAE‑II_x_^+^** |
| pH 5.5 buffer (5.0 vol%) | *n*‑propylamine | slow hydrolysis of **DAE‑II_x_^+^** |
| AcOH (0.1 vol%) | **Oct‑NH_2_**, 5‑trifluoromethylindoline | no reaction |
| TFA (0.1 vol%) | **Oct‑NH_2_**, 5‑trifluoromethylindoline | no reaction |
| - | 5‑trifluoromethylindoline | no reaction |
| - | **Oct‑NH_2_**, **TEG‑NH_2_**, ethylene diamine, histamine | amine exchange with **DAE‑II_x_** confirmed |

[a] successful amine exchange was indirectly confirmed by the occurrence of the corresponding inactive aniline derivatives*, e.g.* **DAE‑I_Oct_** or **DAE‑I_TEG_**, resulting from ring-opening of the exchanged species with 450 nm blue light.

Ultraviolet/visible (UV/vis) Spectroscopy

UV/vis spectroscopy was performed using a *LOT-Oriel 500 W Hg(Xe) lamp* attached to a *LOT-Oriel MSH-300 monochromator* with variable slit width and a *LOT-Oriel shutter*. The slit widths were set to 10.00 nm or 20 nm for the exit slit and 10.81 nm or 21.62 nm for the entrance slit. An optical fiber was used to transfer the emitted light to the measuring chamber of a *Cary60 spectrophotometer by Agilent*. The spectrophotometer was equipped with a thermostated *Luma 40 cuvette holder* with a build in magnetic stirrer that was kept at constant temperature with a stirring speed of 1200 rpm. The light beam from the optical fiber was set up orthogonal to the measurement beam of the spectrometer to allow for simultaneous irradiation and acquisition of spectra. 3 mL *quartz cuvettes* (10 mm × 10 mm) or 3 mL *UHV quartz cuvettes* (10 mm × 10 mm) from *Starna GmbH* were used for spectroscopy of solutions. Spectra were measured from 200 nm to 800 nm with a scanning speed of 2400 nm min^−1^ and a resolution of 1 nm. LED irradiation was performed with *mounted LEDs* by *Thorlabs* (*M340L5* for 340 nm, *M365LP1* for 365 nm, *M450LP2* for 450 nm, and *M590L4* for 590 nm) with typical output powers of 69.2 – 3041.5 mW and a FWHM between 9 and 18 nm powered by an *LEDD1B T‑cube LED driver*. LED light was focused using an *SM2F32‑A* *adjustable collimator adapter* equipped with an *ACL50832U* (for <350 nm) or an *ACL50832U‑A* condenser lens (for 350 – 700 nm). Samples for spectroscopy were prepared using a *Cubis MCE3.6P-2S00-M* microbalance by *Sartorius* with a scale interval of 1 μg and spectroscopy grade solvents that were filtered through a 0.2 μm syringe-driven PTFE filter for MeOH solutions and a PA filter for aqueous solutions. All solutions were sonicated prior to measurement.

Nuclear Magnetic Resonance (NMR) Spectroscopy

^1^H and ^13^C{^1^H} NMR spectra were recorded on a *Bruker Avance III Spectrometer* (Bruker Corporation, Billerica, MA, USA) at 400 MHz or 300 MHz for ^1^H and 100 MHz or 75 MHz for ^13^C, respectively. ^19^F{^1^H} NMR spectra were measured on a *Bruker AV600 Spectrometer* operating at 565 MHz and a *Bruker Avance III Spectrometer* operating at 375 MHz. Chemical shifts are reported to the nearest 0.01 ppm for ^1^H and ^19^F spectra or 0.1 ppm for ^13^C spectra and were referenced to the residual solvent signals in ^1^H (7.26 ppm for CDCl_3_, 5.32 ppm for CH_2_Cl_2_, 3.31 ppm for CD_3_OD, and 2.50 ppm for DMSO‑*d*_6_) and ^13^C spectra (77.2 ppm for CDCl_3_, 53.8 ppm for CD_2_Cl_2_, 49.0 ppm for CD_3_OD, and 39.5 ppm for DMSO‑*d*_6_). Peak forms are abbreviated as s (singlet), d (doublet), dd (doublet of doublets), t (triplet), td (triplet of doublets), q (quartet), p (quintet), dp (doublet of quintets), m (multiplet) , and br (broad).

Ultra-High Performance Liquid Chromatography (UPLC)

Ultra-High performance liquid chromatography coupled to mass spectrometry was performed on a *Waters Acquity I-Class* system using gradient elution with a MeCN/H_2_O mixture (5 – 95% MeCN in H_2_O or 20 – 95% MeCN in H_2_O) containing 0.1 vol% formic acid through a C18 reversed-phase column and detection with a *Waters QDa Mass Detector* and a *Waters Diode Array Detector*.

Medium Performance Liquid Chromatography (MPLC)

Reversed-phase medium performance liquid chromatography was carried out on a *Biotage Isolera One* using *Biotage Sfär C18 Duo* columns.

High-Resolution Mass Spectrometry (HR‑MS)

Mass spectra were recorded by electrospray ionization (ESI) with high resolution mass spectrometry (HR-MS) on a *Bruker micrOTOF Q II* (Bruker Daltonics, Bremen, Germany) with a source voltage of 4.5 kV. Detection was in positive ion mode.

Dynamic Light Scattering (DLS)

DLS measurements were performed on an *ALV/CGS-3 Compact Goniometer System* (ALV-Laser Vertriebsgesellschaft mbH, Hessen, Germany) with an *ALV/LSE 5004 Tau Digital Correlator*. The *JDS Uniphase laser* was used that operates at λ = 632.8 nm. The samples were measured at a fixed scattering angle *θ* = 90°. Temperature trends of the microgels were measured from 10-50 °C in 2 °C steps and the VPTT was determined by sigmoidal Boltzmann fit. For pH dependent measurements, the samples were diluted in the respective buffer solutions ranging from pH 3.2-10.0 with a constant ionic strength of 10 mM and the hydrodynamic diameters were measured at 20 and 50 °C. The ionic strength (*I* = 1-500 mM) measurements were carried out at 20 °C. The intensity time correlation functions were analyzed using cumulant algorithm. All samples were filtered (1.2 µm PET filter, Chromafil®) before the measurements and diluted with HPLC grade water or buffer solution.

Electrophoretic Light Scattering (ELS)

The electrophoretic mobility measurements were carried out with a *Zetasizer Ultra* (Malvern Panalytical Ltd., UK). For all measurements, 100 µL of microgel solution was diluted with buffer solution (*I* = 10 mM) and transferred into a disposable capillary zeta cell cuvette (DTS1070). The measurements were conducted at 20 and 50 °C at a scattering angle *θ* = 12.5° and with an applied voltage of 150 V. The electrophoretic mobility of each sample was analyzed using the software ZS Xplorer.

Bright‑Field Scanning Transmission Electron Microscopy (BFSTEM)

BFSTEM images were captured on an *SU9000 ultrahigh resolution SEM* (Hitachi High-Technologies, Tokyo, Japan). The microgel samples were diluted to a concentration of 1 mg mL^-1^ and a single droplet was placed onto each carbon coated copper grid (300 Mesh Cu) from *Agar Scientific Ltd*. The microgel size of the recorded images were determined with the software ImageJ 1.52t.

Synthesis of Diarylethene Crosslinker DAE‑I_x_

Diarylethene crosslinker **DAE‑I_x_** was synthesized in a modular twelve-step procedure from 2‑methylthiophene, cyclohexanone and 1‑iodo-4-chlorobenzene, as well as 3,3’‑((oxybis(ethane-2,1-diyl))bis(oxy))bis-(propan-1-amine) (**Scheme S1**). Thiophene building block **3** was synthesized by bromination of 2‑methylthiophene followed by *in-situ* borylation and Suzuki coupling with ethyl 4‑iodobenzoate. Chlorobenzene building block **5** was prepared by an α‑arylation of cyclohexanone with 1‑iodo-4-chlorobenzene and subsequent triflation with *N*‑phenylbis(trifluoromethanesulfonimide). Linker amine **8** was synthesized by means of single Boc-protection of 3,3’‑((oxybis(ethane-2,1-diyl))bis(oxy))bis-(propan-1-amine) followed by amidation with methacryloyl chloride and a final Boc-deprotection. **3** and **5** were subsequently Suzuki crosscoupled, the resulting ester **9** was saponified and connected with amine **8** by a 1‑ethyl-3-(3-dimethylaminopropyl)carbodiimide (EDC) coupling. In a final step, chloro-DAE **11** was transferred to crosslinker **DAE‑I_x_** *via* a Buchwald-Hartwig reaction. Overall, following this procedure yielded 6% of the target compound over a total of twelve steps considering the least yielding pathway, *i.e.*, *via* chlorobenzene building block **5**.


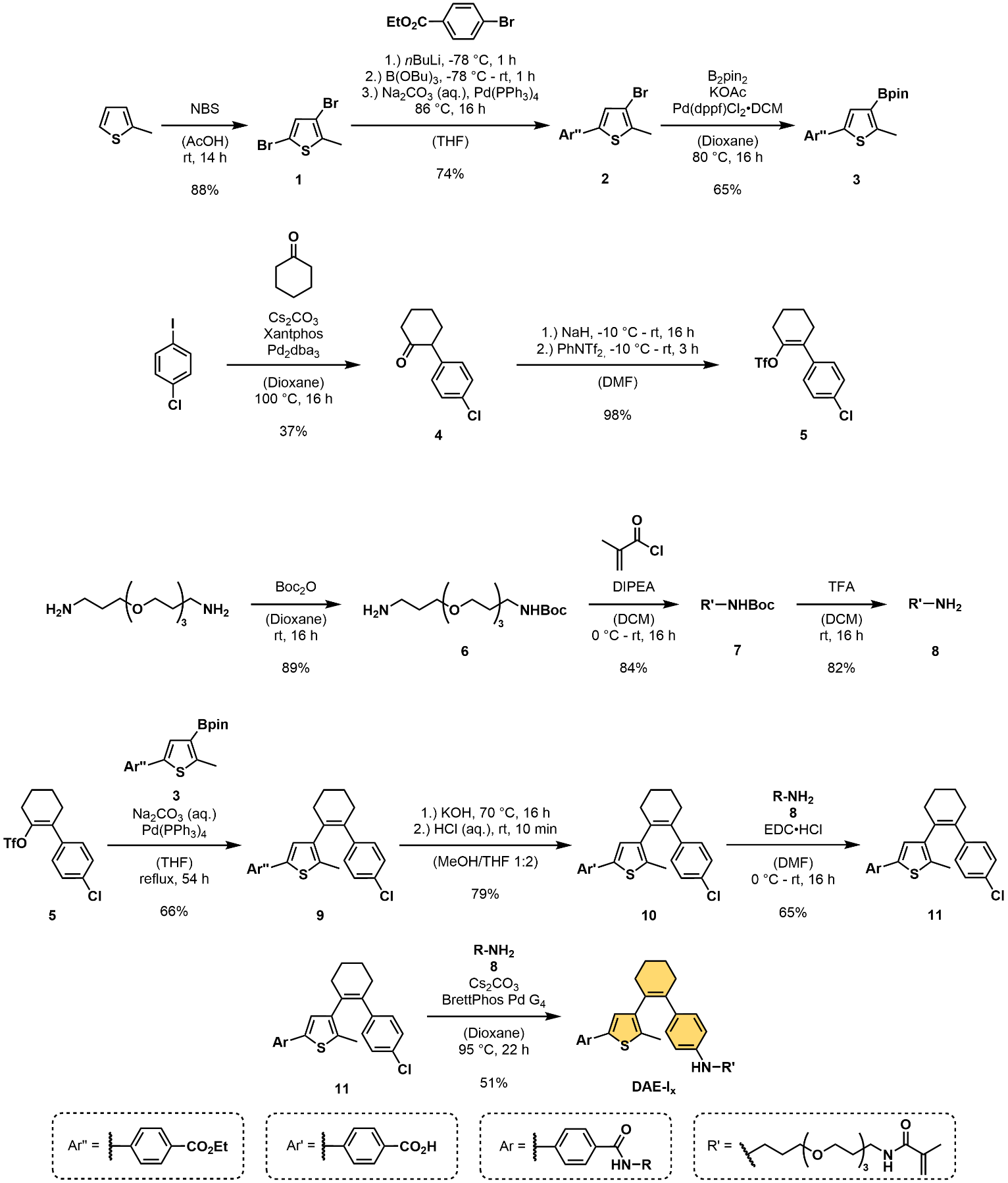


Scheme S1. Twelve-step synthesis of DAE crosslinker DAE‑I_x_.

**3,5-Dibromo-2-methylthiophene (1)**


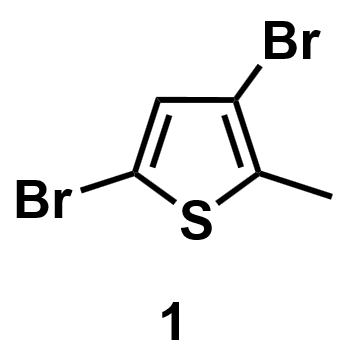


Thiophene **1** was synthesized according to the literature.^[2-4]^ 2‑Methylthiophene (13.5 mL, 139 mmol, 1.0 eq) was dissolved in glacial acetic acid (233 mL). *N*‑Bromosuccinimide (49.6 g, 279 mmol, 2.0 eq) was added and the mixture was stirred at rt for 14 h. 1.0 M NaOH (200 mL) was added and the mixture was extracted with DCM (3x 100 mL). The organic phases were washed with sat. Na_2_S_2_O_3_ solution (3x 100 mL). The organic phase was dried over MgSO_4_ and the solvent was removed under reduced pressure. The crude product was further purified by filtration over silica with cyclohexane as eluent to provide 3,5‑dibromo-2-methylthiophene (**1**) (31.4 g, 123 mmol, 88%) as a yellow oil. The analytic data are in alignment with literature.

**^1^H NMR (400 MHz, CDCl_3_):** δ (ppm) = 6.86 (s, 1H), 2.34 (s, 3H); **^13^C{^1^H} NMR (100 MHz, CDCl_3_):** δ (ppm) = 136.2, 132.1, 108.8, 108.6, 15.0.

**Ethyl 4‑(4-bromo-5-methylthiophen-2-yl)benzoate (2)**


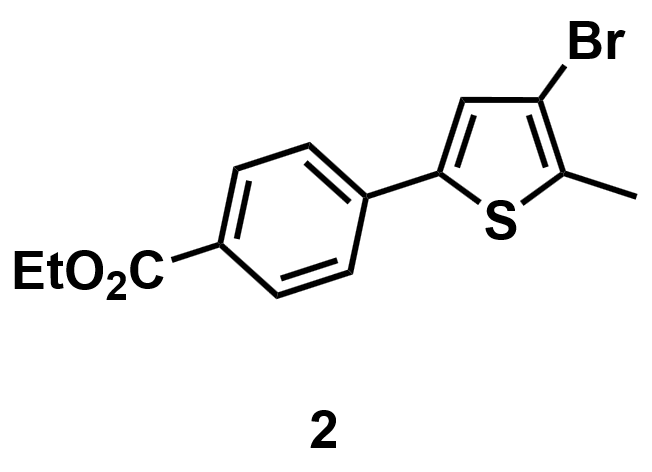


This protocol was adopted from literature.^[2]^ Under an argon atmosphere, thiophene **1** (5.59 g, 21.8 mmol, 1.00 eq.) was dissolved in dry and degassed THF (53 mL) and the solution was cooled to -78 °C. A 2.5 M solution of *n‑*butyllithium in hexanes (8.70 mL, 21.8 mmol, 1.00 eq.) was added dropwise *via* a syringe and the dark red solution was stirred at -78 °C for 30 min while turning pale yellow. The solution was subsequently treated with tributyl borate (5.90 mL, 21.8 mmol, 1.00 eq.) and stirred for 1 h while slowly warming to rt. Distilled water (13 mL), sodium carbonate (6.94 g, 65.5 mmol, 3.00 eq.), ethyl 4‑bromobenzoate (5.00 g, 21.8 mmol, 1.00 eq.) and tetrakis(triphenylphosphine)palladium(0) (757 mg, 655 μmol, 0.03 eq.) were added. The mixture was degassed with argon for 5 min and subsequently stirred at 85 °C for 21 h. EtOAc (100 mL) was added and the reaction was washed with distilled water (100 mL) and brine (100 mL). The organic layer was dried over MgSO_4_, filtered through a pad of celite and the solvent was removed under reduced pressure. The crude product was further purified by flash column chromatography (silica, cyclohexane/EtOAc 95:5) and subsequent recrystallization from *n‑*heptane to provide ethyl 4‑(4‑bromo-5-methylthiophen-2-yl)benzoate (**2**) (5.24 g, 16.1 mmol, 74%) as beige crystals.

**^1^H NMR (400 MHz, CDCl_3_):** δ (ppm)= 8.03 – 8.00 (m, 2H), 7.55 – 7.52 (m, 2H), 7.19 (s, 1H), 4.38 (q, *J* = 7.1 Hz, 2H), 2.42 (s, 2H), 1.40 (t, *J* = 7.1 Hz, 3H); **^13^C{^1^H} NMR (100 MHz, CDCl_3_):** δ (ppm) = 166.2, 139.9, 137.6, 135.4, 130.4, 129.5, 127.0, 124.9, 110.5, 61.1, 15.1, 14.5; **HR‑MS (ESI^+^):** m/z calculated for C_14_H_13_BrO_2_S [M+H]^+^: 324.9892, found: 324.9893.

**Ethyl 4‑(5-methyl-4-(4,4,5,5-tetramethyl-1,3,2-dioxaborolan-2-yl)thiophen-2-yl)benzoate (3)**


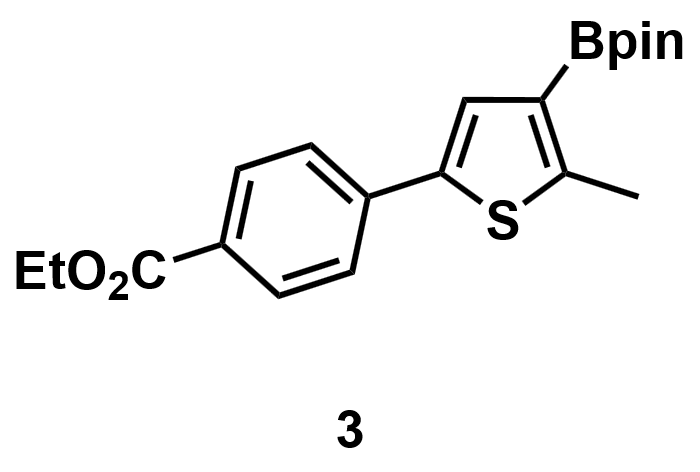


Under an argon atmosphere, thiophene **2** (5.50 g, 16.9 mmol, 1.00 eq.) and bis(pinacolato)diboron (4.72 g, 18.6 mmol, 1.10 eq.) were dissolved in dry 1,4‑dioxane (34 mL). KOAc (3.32 g, 33.8 mmol, 2.00 eq.) was added and the mixture was degassed with argon for 5 min. Dichloro[1,1'‑bis(diphenylphosphino)ferrocene]palladium(II) methylene chloride adduct (691 mg, 846 μmol, 0.05 eq) was added and the mixture was stirred at 80 °C for 16 h. After that time, additional dichloro[1,1'‑bis(diphenylphosphino)ferrocene]palladium(II) methylene chloride adduct (138 mg, 169 μmol, 0.01 eq) and bis(pinacolato)diboron (859 mg, 3.38 mmol, 0.20 eq) were added. The mixture was degassed with argon for 5 min and stirred at 80 °C for an additional 4 h. EtOAc (100 mL) was added, the mixture was filtered through a pad of celite, and the solvent was removed under reduced pressure. The crude product was further purified by flash column chromatography (silica, cyclohexane/EtOAc 98:2 → 95:5) to provide ethyl 4‑(5‑methyl-4-(4,4,5,5-tetramethyl-1,3,2-dioxaborolan-2-yl)thiophen-2-yl)benzoate (**3**) (4.06 g, 10.9 mmol, 65%) as a pale yellow solid.

**^1^H NMR (400 MHz, CDCl_3_):** δ (ppm) = 8.02 – 7.99 (m, 2H), 7.63 – 7.60 (m, 2H), 7.54 (s, 1H), 4.37 (q, *J* = 7.1 Hz, 2H), 2.71 (s, 3H), 1.40 (t, *J* = 7.2 Hz, 3H), 1.34 (s, 12H); **^13^C{^1^H} NMR (100 MHz, CDCl_3_):** δ (ppm) = 166.5, 154.0, 139.7, 138.8, 130.6, 130.2, 128.6, 125.3, 83.6, 61.0, 25.0, 16.2, 14.5; **HR‑MS (ESI^+^):** m/z calculated for C_20_H_25_BO_4_S [M+H]^+^: 373.1639, found: 373.1655.

**2‑(4‑Chlorophenyl)cyclohexan-1-one (4)**


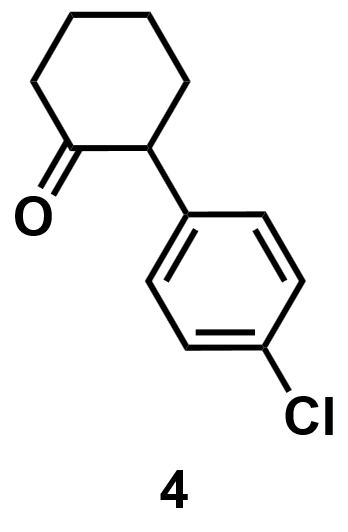


This protocol was adopted from literature.^[5]^ Under an argon atmosphere, 1‑iodo-4-chlorobenzene (20.0 g, 83.9 mmol, 1.00 eq.), Xantphos (971 mg, 1.68 mmol, 0.02 eq.) and cyclohexanone (17.4 mL, 16.5 g, 168 mmol, 2.00 eq) were dissolved in dry 1,4‑dioxane (84.0 mL). Cesium carbonate (60.1 g, 185 mmol, 2.20 eq.) was added and the mixture was degassed with argon for 5 min. Tris(dibenzylideneacetone)dipalladium(0) (768 mg, 839 μmol, 0.01 eq.) was added and the mixture was stirred at 100 °C for 20 h. The reaction was diluted with EtOAc (150 mL), filtered through a pad of celite, and the solvent was removed under reduced pressure. The crude product was purified by flash column chromatography (silica, cyclohexane/EtOAc 8:2) to provide 2‑(4‑chlorophenyl)cyclohexan-1-one (**4**) (6.38 g, 30.6 mmol, 37%) as a pale yellow solid. The analytic data are in alignment with literature.^[6]^

**^1^H NMR (400 MHz, CDCl_3_):** δ (ppm) = 7.32 – 7-28 (m, 2H), 7.09 – 7.05 (m, 2H), 3.59 (dd, *J* = 12.2, 5.4 Hz, 1H), 2.57 – 2.40 (m, 2H), 2.30 – 2.11 (m, 2H), 2.04 – 1.76 (m, 4H); **^13^C{^1^H} NMR (100 MHz, CDCl_3_):** δ (ppm) = 209.9, 137.3, 132.8, 130.0, 128.6, 56.9, 42.3, 35.4, 27.9, 25.5. **HR‑MS (ESI^+^):** m/z calculated for C_12_H_13_ClO [M+H]^+^: 209.0728, found: 209.0728.

**4’‑Chloro-3,4,5,6-tetrahydro-[1,1’-biphenyl]-2-yl trifluoromethanesulfonate (5)**


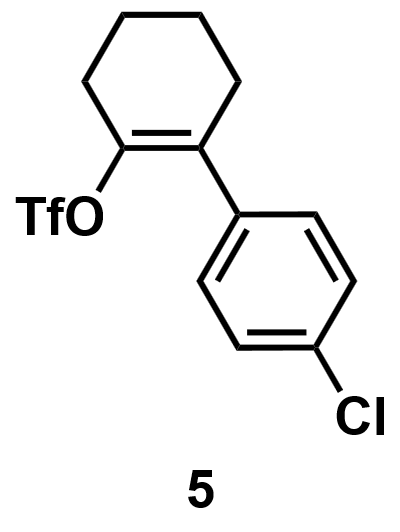


This protocol was adopted from literature.^[7-8]^ Under an argon atmosphere, ketone **4** (7.20 g, 34.5 mmol, 1.0 eq.) was dissolved in dry DMF (258 mL). The solution was cooled to ‑10 °C and a 60 wt% dispersion of sodium hydride in mineral oil (1.79 g, 44.9 mmol, 1.3 eq.) was added in four portions over 30 min. The solution was stirred for 4 h at -10 °C until no further gas formation could be observed, after which it was stirred for 16 h while warming to rt. After that time, the solution was cooled to -10 °C again and *N*‑phenylbis(trifluoromethanesulfonimide) (13.6 g, 38.0 mmol, 1.1 eq.) was added. The solution was stirred at -10 °C for 2 h at the same temperature and an additional 2 h while warming to room temperature. The reaction was quenched by the addition of distilled water (10 mL). EtOAc (500 mL) was added, the organic phase was washed with brine (500 mL) and distilled water (500 mL), dried over MgSO_4_, and the solvent was removed under reduced pressure. The crude product was further purified by filtration through a pad of silica (cyclohexane/EtOAc 9:1) to provide 4’‑chloro-3,4,5,6-tetrahydro-[1,1’-biphenyl]-2-yl trifluoromethanesulfonate (**5**) (11.5 g, 33.7 mmol, 98%) as a colorless oil.

**^1^H NMR (400 MHz, CDCl_3_):** δ (ppm) = 7.35 – 7.32 (m, 2H), 7.21 – 7.18 (m, 2H), 2.52 – 2.41 (m, 4H), 1.92 – 1.72 (m, 4H); **^13^C{^1^H} NMR (100 MHz, CDCl_3_):** δ (ppm) = 144.3, 135.5, 134.0, 130.1, 129.6, 128.7, 188.2 (q, *J* = 319.85 Hz, CF_3_), 31.3, 28.2, 23.0, 22.1; **^19^F{^1^H} NMR (375 MHz, CDCl_3_):** δ (ppm) = -75.10; **HR‑MS (ESI^+^):** *m/z* calculated for C_13_H_12_ClF_3_O_3_S [M+Na]^+^: 363.0040, found: 363.0057.

***tert‑*Butyl (3‑(3‑aminopropoxy)-2-(2-ethoxyethoxy)propyl)carbamate (6)**


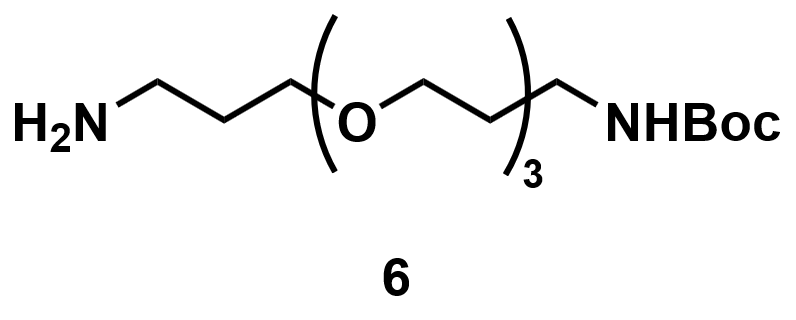


This protocol was adopted from literature.^[9]^ A solution of di‑*tert*-butyl dicarbonate (24.7 g, 113 mmol, 1.0 eq) in 1,4‑dioxane (170 mL) was slowly dropped into neat 3,3’‑((oxybis(ethane-2,1-diyl))bis(oxy))bis-(propan-1-amine) (199 g, 904 mmol, 8.0 eq.) and stirred at rt for 16 h. The solvent was removed under reduced pressure and the residue was redissolved in distilled water (500 mL) to which a sat. sodium bicarbonate solution (50 mL)was added. The aqueous phase was extracted with dichloromethane (3x 500 mL), the combined organic layers were dried over MgSO_4_, and the solvent was removed under reduced pressure to provide *tert‑*butyl (3‑(3‑aminopropoxy)-2-(2-ethoxyethoxy)propyl)carbamate (**6**) (32.3 g, 101 mmol, 89%) as a pale yellow oil that was used in the next step without further purification. The analytic data are in good alignment with literature.^[10]^

**^1^H NMR (300 MHz, DMSO-*d*_6_):** δ (ppm) = 6.75 (t, *J* = 6.0 Hz, 1H), 3.52 – 3.32 (m, 12H), 3.25 – 3.14 (m, 1H), 2.95 (q, *J* = 6.9 Hz, 2H), 2.56 (t, *J* = 6.9 Hz) and 2.39 (t, *J* = 7.2 Hz, 1H), 1.59 (p, *J* = 6.4 Hz, 4H), 1.37 (s, 11H); **^13^C{^1^H} NMR (75 MHz, DMSO‑*d*_6_):** δ (ppm) = 155.5, 77.4, 73.9, 69.8, 69.5, 68.6, 68.5, 68.1, 54.9, 37.2, 33.4, 29.7, 28.2, 27.4; **HR‑MS (ESI^+^):** *m/z* calculated for C_15_H_32_N_2_O_5_ [M+H]^+^: 321.2384, found: 321.4202.

***tert*‑Butyl (16‑methyl-15-oxo-4,7,10-trioxa-14-azaheptadec-16-en-1-yl)carbamate (7)**


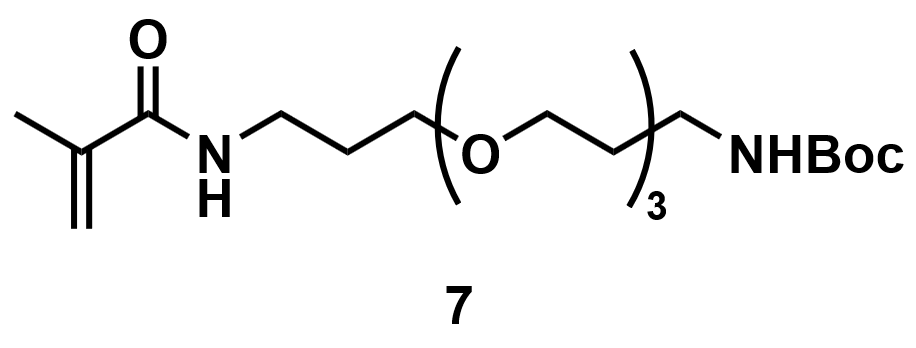


A solution of Boc‑protected amine **6** (15.0 g, 46.8 mmol, 1.0 eq.) and DIPEA (13.0 mL, 74.9 mmol, 1.6 eq.) in DCM (156 mL) was cooled in an ice-bath and methacryloyl chloride (6.30 mL, 65.5 mmol, 1.4 eq.) was added dropwise. The mixture was stirred for 16 h while warming to rt, after which it was washed with a sat. sodium bicarbonate solution (3x 150 mL). The organic layer was dried over MgSO_4_ and the solvent was removed under reduced pressure. The resulting crude product was purified by flash column chromatography (DCM/MeOH 98:2) to provide *tert*‑butyl (16‑methyl-15-oxo-4,7,10-trioxa-14-azaheptadec-16-en-1-yl)carbamate (**7**) (15.3 g, 39.3 mmol, 84%) as a pale yellow oil to which a small amount (spatula’s tip) of 2,6-di-*tert*-butyl-4-methylphenol (BHT) was added.

**^1^H NMR (400 MHz, DMSO-*d*_6_):** δ (ppm) = 7.86 (t, *J* = 6.0 Hz, 1H), 6.75 (t, *J* = 5.6 Hz, 1H), 5.62 (s, 1H), 5.30 (t, *J* = 1.6 Hz, 1H), 3.54 – 3.35 (m, 12H), 3.15 (td, *J* = 7.0, 5.7 Hz, 2H), 2.95 (q, *J* = 6.6 Hz, 2H), 1.84 (t, *J* = 1.4 Hz, 3H), 1.66 (p, *J* = 6.7 Hz, 2H), 1.58 (p, *J* = 6.6 Hz, 2H), 1.37 (s, 9H); **^13^C{^1^H} NMR (100 MHz, DMSO-*d*_6_):** δ (ppm) = 167.4, 155.6, 140.0, 118.8, 77.4, 69.8, 69.8, 69.6, 69.5, 68.3, 68.1, 37.2, 36.4, 29.7, 29.3, 28.3, 18.6; **HR‑MS (ESI^+^):** *m/z* calculated for C_19_H_36_N_2_O_6_ [M+H]^+^: 389.2646, found: 389.2655.

***N‑*(3‑(2‑(2‑(3‑Aminopropoxy)ethoxy)ethoxy)propyl)methacrylamide (8)**


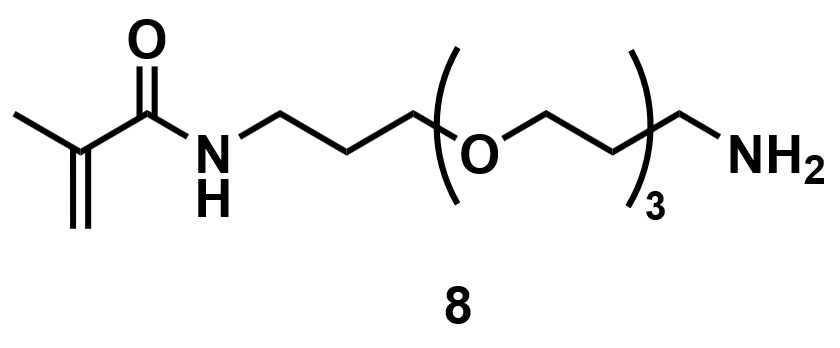


Boc‑protected methacrylamide **7** (10.7 g, 24.8 mmol, 1.00 eq.) was treated with TFA (15.5 mL, 202 mmol, 8.17 eq.) and stirred at rt for 16 h. The mixture was dropped into ice-cooled 1.0 M NaOH solution (200 mL, 203 mmol, 8.20 eq.) and the resulting aqueous phase was subsequently extracted with DCM (3x 200 mL). The combined organic phases were dried over MgSO_4_ and the solvent was removed under reduced pressure. The resulting crude product was purified by flash column chromatography (silica, DCM/MeOH 3:1 with 1.0 vol% Et_3_N) to provide *N‑*(3‑(2‑(2‑(3‑aminopropoxy)ethoxy)ethoxy)propyl)methacrylamide (**8**) (5.84 g, 20.2 mmol, 82%) as a pale yellow oil to which a small amount (spatula’s tip) of BHT was added.

**^1^H NMR (400 MHz, DMSO-*d*_6_):** δ (ppm) = 7.91 (t, *J* = 5.7 Hz, 1H), 5.63 (t, *J* = 1.2 Hz, 1H), 5.30 (p, *J* = 1.6 Hz, 1H), 3.55 – 3.36 (m, 12H), 3.15 (td, *J* = 7.0, 5.7 Hz, 2H), 2.57 (t, *J* = 6.8 Hz, 2H), 2.34 (s, br, 2H), 1.84 (s, 3H), 1.66 (p, *J* = 6.7 Hz, 2H), 1.56 (p, *J* = 6.6 Hz, 2H); **^13^C{^1^H} NMR (100 MHz, DMSO-*d*_6_):** δ (ppm) = 167.4, 140.1, 118.8, 69.8, 69.6, 69.5, 68.4, 68.4, 38.7, 36.4, 33.0, 29.3, 18.7; **HR‑MS (ESI^+^):** *m/z* calculated for C_14_H_28_N_2_O_4_ [M+H]^+^: 289.2122, found: 289.2147.

**Ethyl 4‑(4‑(4'‑chloro-3,4,5,6-tetrahydro-[1,1'-biphenyl]-2-yl)-5-methylthiophen-2-yl)benzoate (9)**

**
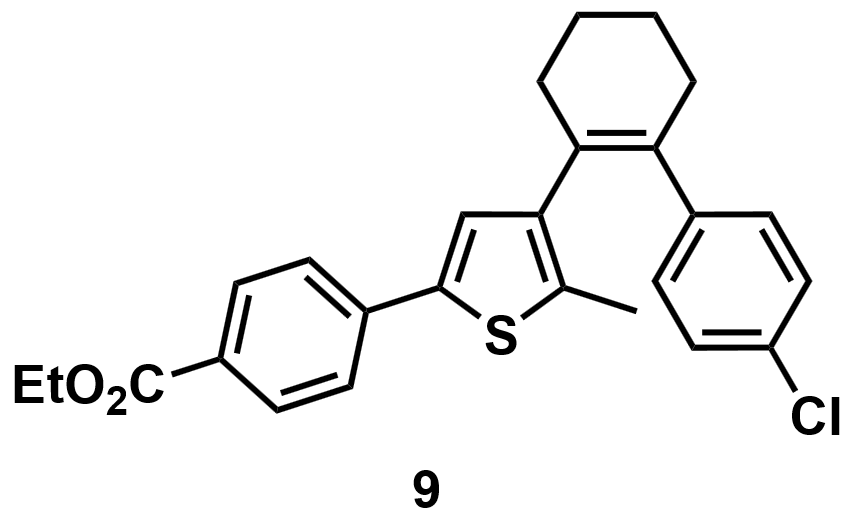
**

Under an argon atmosphere, boronic ester **3** (4.00 g, 10.7 mmol, 1.00 eq.) and triflate **5** (3.66 g, 10.7 mmol, 1.00 eq) were dissolved in THF (107 mL). Distilled water (16 mL) and Na_2_CO_3_ (3.42 g, 32.2 mmol, 3.00 eq.) were added and the mixture was degassed with argon for 5 min. Tetrakis(triphenylphosphine)palladium(0) (621 mg, 537 μmol, 0.05 eq.) was added and the mixture was refluxed for 16 h. EtOAc (200 mL) was added and the organic phase was washed with water (200 mL) and brine (200 mL). The organic layer was dried over MgSO_4_ and the solvent was removed under reduced pressure. The crude product was purified by reverse-phase MPLC (C18 silica, MeCN/H_2_O 8:2 → 95:5) to provide ethyl 4‑(4‑(4'‑chloro-3,4,5,6-tetrahydro-[1,1'-biphenyl]-2-yl)-5-methylthiophen-2-yl)benzoate (**9**) (3.08 g, 7.04 mmol, 66%) as an orange oil.

**^1^H NMR (400 MHz, CDCl_3_):** δ (ppm) = 8.02 – 7.98 (m, 2H), 7.56 – 7.53 (m, 2H), 7.14 (s, 1H), 7.10 – 7.08 (m, 2H), 6.97 – 6.94 (m, 2H), 4.38 (q, *J* = 7.1 Hz, 2H), 2.49 – 2.41 (m, 2H), 2.38 – 2.30 (m, 2H), 1.89 (s, 3H), 1.87 – 1.77 (m, 4H), 1.40 (t, *J* = 7.1 Hz, 3H); **^13^C{^1^H} NMR** **(100 MHz, CDCl_3_):** δ (ppm) = 166.5, 142.0, 141.6, 138.8, 138.7, 135.8, 135.2, 131.7, 130.5, 130.3, 129.6, 128.6, 128.0, 126.2, 124.8, 61.1, 32.1, 31.2, 23.4, 23.1, 14.5, 14.2; **HR‑MS (ESI^+^):** *m/z* calculated for C_26_H_25_ClO_2_S [M+H]^+^: 437.1337, found: 437.1359.

**4‑(4‑(4’‑Chloro-3,4,5,6-tetrahydro-[1,1’-biphenyl]-2-yl)-5-methylthiophen-2-yl)benzoic acid (10)**


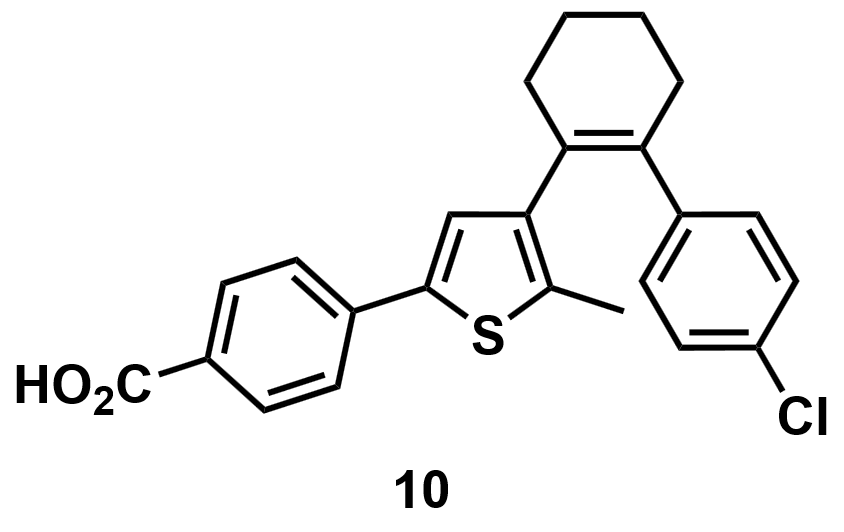


This protocol was adopted from literature.^[11]^ Benzoate **9** (3.00 g, 6.87 mmol, 1.00 eq.) was dissolved in THF (20 ml) and a 2.0 M solution of potassium hydroxide in MeOH (35.2 mL, 70.4 mmol, 10.3 eq.) was added. The solution was heated to 70 °C for 3 h, after which it was concentrated under reduced pressure and treated with 1.0 M hydrochloric acid (35.2 mL, 70.4 mmol, 10.3 eq.). The resulting precipitate was filtered and washed with EtOH to provide 4‑(4‑(4’‑chloro-3,4,5,6-tetrahydro-[1,1’-biphenyl]-2-yl)-5-methylthiophen-2-yl)benzoic acid (**10**) (2.21 g, 5.39 mmol, 79%) as a colorless powder that was used for the next step without further purification.

**^1^H NMR (400 MHz, DMSO-*d*_6_):** δ (ppm) = 12.93 (s, 1H), 7.92 – 7.90 (m, 2H), 7.67 – 7.64 (m, 2H), 7.48 (s, 1H), 7.21 – 7.18 (m, 2H), 7.07 – 7.03 (m, 2H), 2.46 – 2.38 (m, 2H), 2.35 – 2.27 (m, 2H), 1.85 (s, 3H), 1.82 – 1.71 (m, 4H); **^13^C{^1^H} NMR (100 MHz, DMSO-*d*_6_):** δ (ppm) = 166.9, 142.0, 141.5, 137.8, 137.6, 135.2, 134.2, 130.5, 130.1, 130.0, 129.8, 128.9, 127.7, 126.9, 124.5, 31.3, 30.5, 22.7, 22.4, 13.7; **HR‑MS (ESI^+^):** *m/z* calculated for C_24_H_21_ClO_2_S [M-H]^-^: 407.0878, found: 407.0891.

**4‑(4‑(4’‑Chloro-3,4,5,6-tetrahydro-[1,1’-biphenyl]-2-yl)-5-methylthiophen-2-yl)-*N*-(16-methyl-15-oxo-4,7,10-trioxa-14-azaheptadec-16-en-1-yl)benzamide (11)**


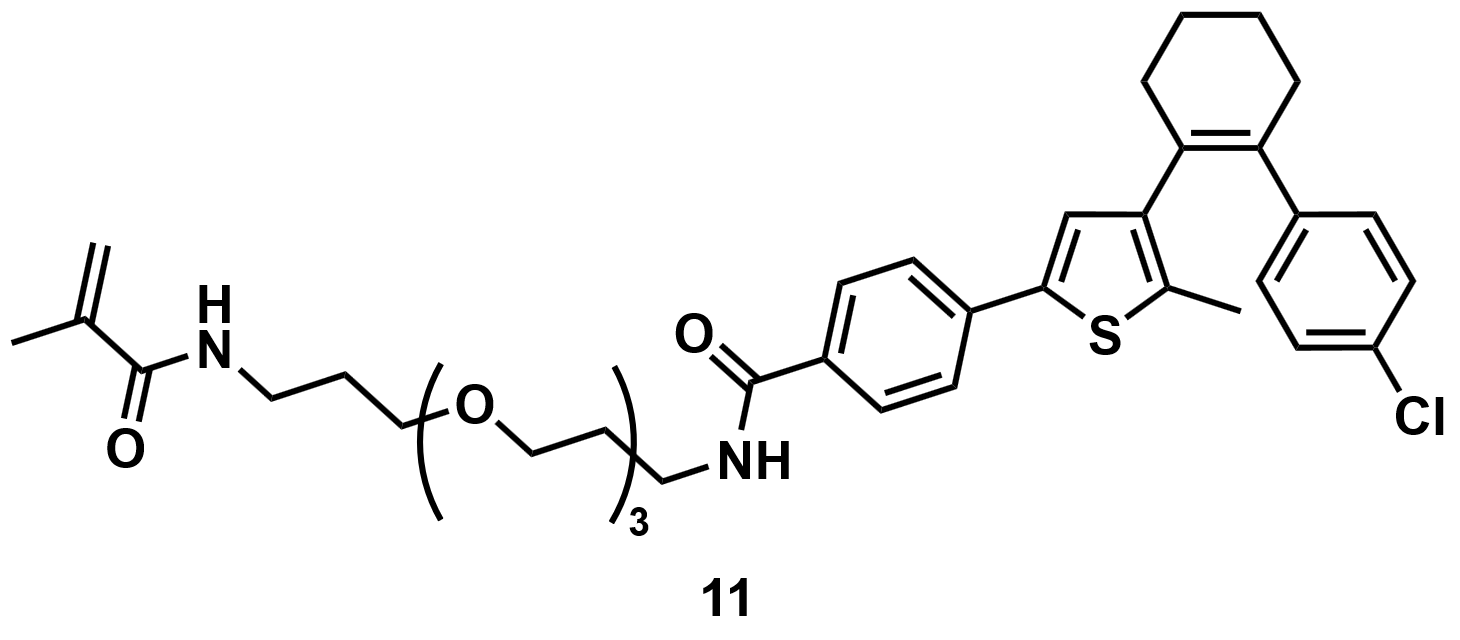


This protocol was adopted from literature.^[12]^ Under an argon atmosphere, benzoic acid **10** (620 mg, 1.52 mmol, 1.00 eq.) was dissolved in dry DMF (23.3 mL). EDC·HCl (320 mg, 1.67 mmol, 1.10 eq.) and DMAP (41.5 mg, 340 μmol, 0.22 eq.) were added and the solution was cooled to 0 °C. Amine **16** (481 mg, 1.67 mmol, 1.10 eq.) was added dropwise and the solution was stirred for 16 h while warming to rt. The reaction was diluted with EtOAc (100 mL) and washed with a sat. sodium bicarbonate solution (100 mL). The aqueous phase was extracted with EtOAc (100 mL), the combined organic layers were dried over MgSO_4_, and the solvent was removed under reduced pressure. The resulting crude product was purified by flash column chromatography (silica, DCM/MeOH 95:5). To remove residual DMF, the purified product was redissolved in EtOAc (100 mL) and washed again with water (100 mL) and brine (100 mL). The organic phase was dried over MgSO_4_ and the solvent was removed under reduced pressure to provide 4‑(4‑(4’‑chloro-3,4,5,6-tetrahydro-[1,1’-biphenyl]-2-yl)-5-methylthiophen-2-yl)-*N*-(16-methyl-15-oxo-4,7,10-trioxa-14-azaheptadec-16-en-1-yl)benzamide (**11**) (676 mg, 994 μmol, 65%) as a pale orange oil to which small amount (spatula’s tip) of BHT was added.

**^1^H NMR (400 MHz, CD_2_Cl_2_):** δ (ppm) = 7.78 – 7.75 (m, 2H), 7.56 – 7.53 (m, 2H), 7.13 – 7.08 (m, 4H), 7.13 – 6.98 (m, 2H), 6.55 (s, 1H), 5.65 (t, *J* = 1.1 Hz, 1H), 5.27 (p, *J* = 1.6 Hz, 1H), 3.66 – 3.47 (m, 14H), 3.34 (q, *J* = 6.2 Hz, 2H), 2.48 – 2.40 (m, 2H), 2.38 – 2.30 (m, 2H), 1.91 (s, 6H), 1.89 – 1.79 (m, 6H), 1.78 – 1.73 (m, 2H); **^13^C{^1^H} NMR (100 MHz, CD_2_Cl_2_):** δ (ppm) = 168.2, 166.6, 142.7, 142.0, 140.8, 138.9, 137.5, 136.1, 135.0, 133.5, 131.8, 131.0, 130.0, 128.1, 127.9, 126.2, 125.1, 119.1, 70.9, 70.7, 70.7, 70.6, 39.2, 38.7, 32.3, 31.5, 29.5, 29.4, 23.6, 23.3, 18.8, 14.1; **HR‑MS (ESI^+^):** *m/z* calculated for C_38_H_47_ClN_2_O_5_S [M+H]^+^: 679.2967, found: 679.2989.

***N*‑(2‑Methyl-3-oxo-7,10,13-trioxa-4-azahexadec-1-en-16-yl)-4-(5-methyl-4-(4’-((16-methyl-15-oxo-4,7,10-trioxa-14-azaheptadec-16-en-1-yl)amino)-3,4,5,6-tetrahydro-[1,1’-biphenyl]-2-yl)thiophen-2-yl)benzamide (DAE‑I_x_)**


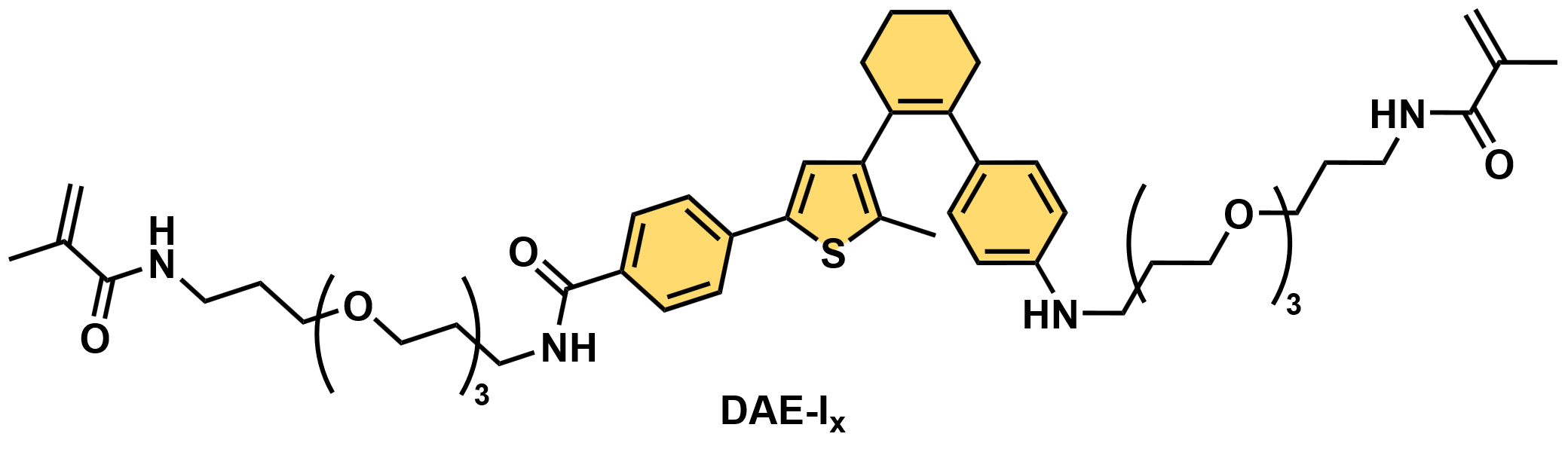


This protocol was adopted from literature.^[13]^ Under an argon atmosphere, chlorobenzene **11** (475 mg, 622 μmol, 1.0 eq.) was dissolved in dry 1,4‑dioxane (7.5 mL). Amine **16** (183 mg, 635 μmol, 1.0 eq.) and cesium carbonate (608 mg, 1.87 μmol, 3.0 eq) were added and the solution was degassed with argon for 5 min. BrettPhos Pd G4 (61.8 mg, 67.1 µmol, 0.1 eq.) was added and the solution was stirred at 95 °C for 22 h. The mixture was diluted with EtOAc (100 mL), filtered through a pad of celite, and the solvent was removed under reduced pressure. The resulting crude product was purified by flash column chromatography (silica, DCM/MeOH 98:2 → 96:4). to provide

*N*‑(2‑methyl-3-oxo-7,10,13-trioxa-4-azahexadec-1-en-16-yl)-4-(5-methyl-4-(4’-((16-methyl-15-oxo-4,7,10-trioxa-14-azaheptadec-16-en-1-yl)amino)-3,4,5,6-tetrahydro-[1,1’-biphenyl]-2-yl)thiophen-2-yl)benzamide (**DAE‑I_x_**) (293 mg, 315 μmol, 51%) as a pale orange oil to which a small amount (spatula’s tip) of BHT was added.

**^1^H NMR (300 MHz, CD_2_Cl_2_):** δ (ppm) = 7.78 – 7.73 (m, 2H), 7,57 – 7.53 (m, 2H), 7.23 (t, *J* = 5.5 Hz, 1H), 7.17 (s, 1H), 6.86 – 6.81 (m, 2H), 6.65 (s, br, 2H), 6.39 – 6.34 (m, 2H), 5.66 – 5-64 (m, 2H), 5.26 (dp, *J* = 3.1, 1.5 Hz, 2H), 4.03 (s, br, 1H), 3.63 – 3.49 (m, 26H), 3.34 (q, *J* = 6.0 Hz, 4H), 3.11 (t, *J* = 6.5 Hz, 2H), 2.44 – 2.40 (m, 2H), 2.33 – 2.29 (m, 2H), 1.91 – 1.70 (m, 28H, overlap with H_2_O peak); **^13^C{^1^H} NMR (75 MHz, CD_2_Cl_2_):** δ (ppm) = 168.3, 168.2, 166.9, 147.2, 143.0, 140.7, 138.2, 137.7, 134.8, 133.2, 132.5, 129.3, 128.1, 127.9, 126.6, 125.0, 119.3, 119.2, 112.1, 70.8 – 70.5 (m), 70.1 42.1, 39.0, 38.7, 38.6, 32.4, 31.7, 29.6, 29.5, 29.4, 29.3, 23.9, 23.5, 18.8, 14.2; **HR‑MS (ESI^+^):** *m/z* calculated for C_52_H_74_N_4_O_9_S [M+H]^+^: 931.5249, found: 931.5288.

Synthesis of Photoswitchable MG‑DAE‑I_x_ Microgels

The **MG‑DAE‑I_x_** microgels with various **DAE‑I_x_** contents (1, 2, and 3 mol%) in the core were synthesized using batch free radical precipitation polymerization in an amber glass flask. VCL (amounts see **Table S2**) and **BIS** (amounts see **Table S2**) were dissolved in water (90 vol%, 34.5 mL). In addition, a solution consisting of **DAE-I_x_** (amounts see **Table S2**) and DMSO (10 vol%, 4 mL) was added. Then, this solution was purged with nitrogen for 1 h at 70 °C under vigorous stirring. For initiation, AMPA (0.020 mmol, 0.8 mol%) was dissolved in water (1 mL) and added to the reaction mixture. Subsequently, the mixture was stirred for 2 h at 70 °C and 250 rpm. The microgels obtained were first dialyzed for 1 d against DMSO (MWCO: 12 – 14 kDa) and then for 4 d against deionized water (MWCO: 12 – 14 kDa) in the dark. The same procedure was used to prepare the PVCL reference microgel (**MG‑PVCL**) without **DAE‑I_x_** and only **BIS** as crosslinker (**Table S2**). The subsequent dialysis was carried out in daylight.

Table S2*.* Amounts of used VCL, BIS, and DAE‑I_x_ for microgel synthesis and the corresponding gravimetrically determined yields.

| Sample | *m*_VCL_ (mg) | *m*_BIS_ (mg) | *n*_BIS_ (µmol) | *m*_DAE-Ix_ (mg) | *n*_DAE-Ix_ (µmol) | yield (%) |
| --- | --- | --- | --- | --- | --- | --- |
| MG-DAE‑I_x_ 1 mol% | 348.2 | 7.7 | 50.0 | 23.3 | 25.0 | 70 |
| MG-DAE‑I_x_ 2 mol% | 348.0 | 4.1 | 25.0 | 46.7 | 50.0 | 68 |
| MG-DAE‑I_x_ 3 mol% | 348.2 | - | - | 69.8 | 75.0 | 62 |
| MG-PVCL | 348.1 | 11.7 | 75.0 | - | - | 79 |

Table S3. Content of DAE‑I_x_ in the corresponding MG‑DAE‑I_x_ microgels determined by UV/vis spectroscopy.

| Sample | Target Content (mol%) | Incorporated Content (mol%)^[a]^ |
| --- | --- | --- |
| MG-DAE‑I_x_ 1 mol% | 1.00 | 0.85 ± 0.03 |
| MG-DAE‑I_x_ 2 mol% | 2.00 | 2.08 ± 0.08 |
| MG-DAE‑I_x_ 2 mol% | 3.00 | 2.42 ± 0.10 |

[a] The error given results from a 4% standard deviation in the extinction coefficient of **DAE‑I_x_** at 314 nm, which exceeds the standard deviation (2 – 3%) of the three-fold determined absorbance of **DAE‑I_x_** in the respective microgels.

General Procedure for Amine Exchange within DAE-Crosslinked Microgels

The respective microgel (10 mg of **MG‑DAE‑I_x_ 1 mol%**, 5 mg of **MG‑DAE‑I_x_ 2 mol%**, or **MG‑DAE‑I_x_ 3 mol%**) was dissolved in methanol (10 mL) and irradiated with 365 nm UV light at 20 °C until no further spectral changes were observed by UV/vis measurements (90 – 120 s). Afterwards, the respective amine (0.1 vol%, 200 eq. with respect to **DAE‑I_x_**) was added and the mixture was stirred at 20 °C for 16 h. The solution was then irradiated at 450 nm until no further changes were observed by UV/vis measurements. The samples were dialyzed for 2 d against methanol (MWCO: 12-14 kDa) and 2 d against deionized water (MWCO: 12‑14 kDa) in the dark. The **MG‑DAE‑I_BF_** samples were furthermore dialyzed for another 1 d against methanol (MWCO: 12‑14 kDa) in the dark.

General Procedure for Light-Gated BF‑NH_2_ Release

The respective **MG‑DAE‑I_BF_** microgel (10 mg of **MG‑DAE‑I_BF_ 1 mol%**, 5 mg of **MG‑DAE‑I_BF_ 2 mol%**, or **MG‑DAE‑I_BF_ 3 mol%**) was dissolved in methanol (10 mL) and irradiated with 365 nm UV light at 20 °C for 60 s. Afterwards, **Oct‑NH_2_** (0.1 vol%) was added and the mixture was stirred at 20 °C for 16 h. The solution was then irradiated at 450 nm for 15 min. The samples were dialyzed for 2 d against methanol (MWCO: 12-14 kDa), and 2 d against deionized water (MWCO: 12‑14 kDa), and an additional 1 d against methanol (MWCO: 12-14 kDa) in the dark.

Photoisomerization of DAE‑I_x_ and Fatigue Measurements


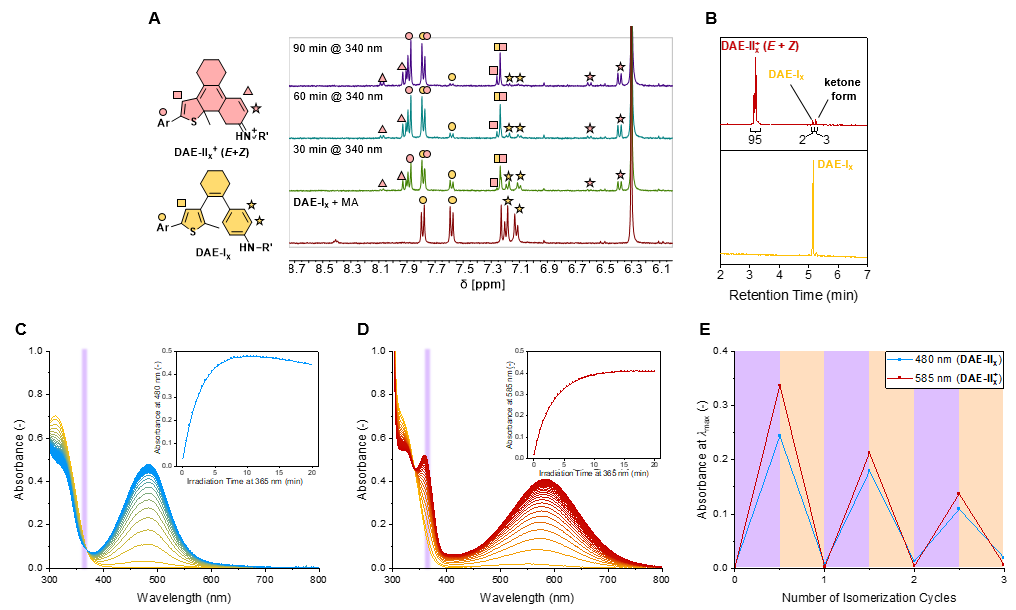


Figure S1. A) ^1^H NMR spectra of DAE‑I_x_ (1.5 mM in deuterated methanol) in the presence of maleic acid (4.6 eq., singlet at 6.3 ppm) before (bottom) and after (top) irradiation with 340 nm UV light for 90 min showing 95% conversion to the *E* and *Z* isomer of DAE‑II_x_^+^ in the PTSS. B) UPLC traces of a 30 µM methanol solution of DAE‑I_x_ before (bottom) and after (top) 365 nm UV light irradiation showing an overall conversion of 98% to DAE‑II_x_^+^ (*E* and *Z*) in the PTSS. The absence of the imine form (DAE‑II_x_) can be explained by the 0.1 vol% content of formic acid and the occurrence of the hydrolyzed ketone form (3%) by the large water content of up to 95 vol% in the eluent mixture. C) UV/vis evolution spectra of the prolonged irradiation of a 30 µM pH 10 buffered (5.0 vol%) methanol solution of DAE‑I_x_ showing the formation of solely imine DAE‑II_x_ at basic pH values. The insert shows the absorbance changes at 480 nm over the course of the 20 min irradiation time. D) UV/vis evolution spectra of the prolonged irradiation of a 30 µM pH 4.0 buffered (5.0 vol%) methanol solution of DAE‑I_x_ showing the formation of solely iminium DAE‑II_x_^+^ at acidic pH values. The insert shows the absorbance changes at 585 nm over the course of the 20 min irradiation time. E) Absorbance of DAE‑II_x_ and DAE‑II_x_^+^ during three consecutive photoisomerization cycles in neutral methanol. Ring-closure was induced by 365 nm and ring-opening by 590 nm irradiation. The measurements displayed in C‑E confirm a facilitated photofatigue reaction of the more electron-rich imine form, *i.e.* DAE‑II_x_.

Content Determination of DAE‑I_x_ in the Synthesized Microgels


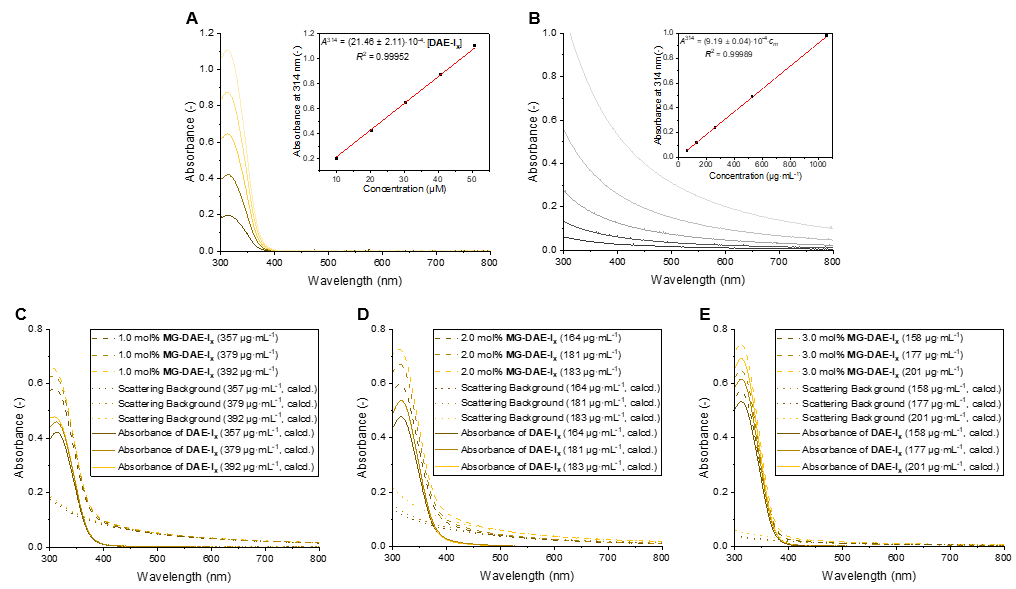


Figure S2. A) Absorbance of DAE‑I_x_ at varying concentrations between 10 – 50 µM revealing a linear correlation. From this data, the extinction coefficient at 314 nm was calculated using Beer’s law^[14-15]^ giving a value of 20900 ± 900 L mol^‑1^ cm^‑1^. B) Scattering of MG‑PVCL at varying concentrations between 50 – 1050 µg mL^‑1^ showing a linear correlation. C) Measured extinction of MG‑DAE‑I_x_ with targeted 1 mol% DAE‑I_x_ (dashed lines), calculated MG‑PVCL scattering background (dotted lines), and corrected absorbance of DAE‑I_x_ within the microgel (solid lines) at three different concentrations (given in the legend). D) Measured extinction of MG‑DAE‑I_x_ with targeted 2 mol% DAE‑I_x_ (dashed lines), calculated MG‑PVCL scattering background (dotted lines), and corrected absorbance of DAE‑I_x_ within the microgel (solid lines) at three different concentrations (given in the legend). E) Measured extinction of MG‑DAE‑I_x_ with targeted 3 mol% DAE‑I_x_ (dashed lines), calculated MG‑PVCL scattering background (dotted lines), and corrected absorbance of DAE‑I_x_ within the microgel (solid lines) at three different concentrations (given in the legend).

BFSTEM Images of Photoswitchable Microgels


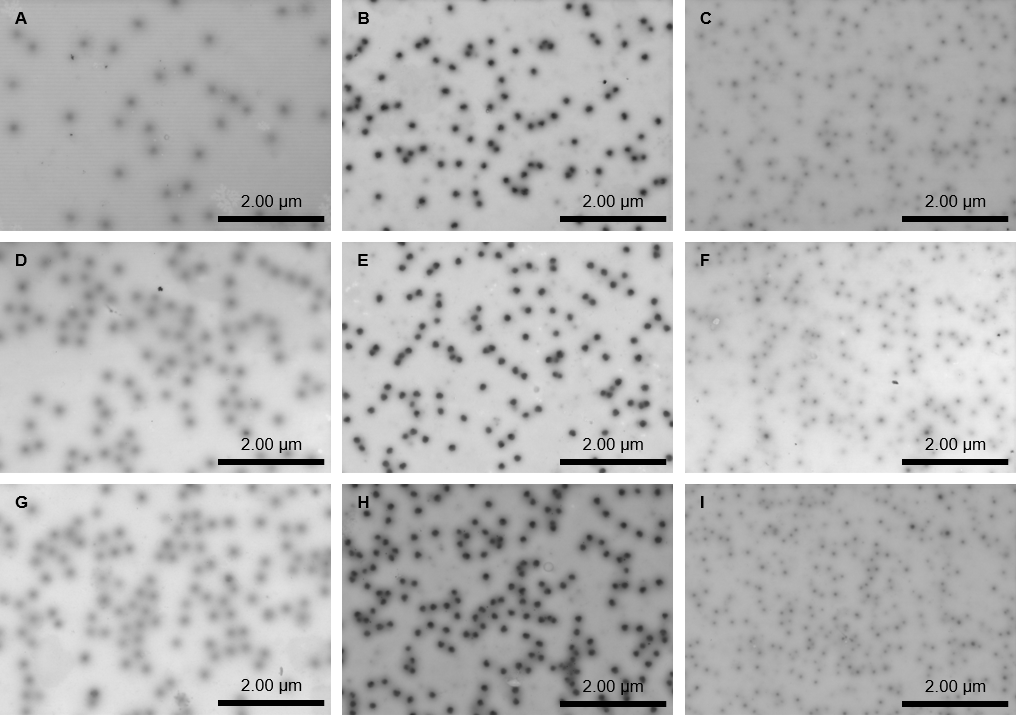


Figure S3. BFSTEM images of MG‑DAE‑I_x_ microgels before (A‑C) and after amine exchange with Oct‑NH_2_ to MG‑DAE‑I_Oct_ (D‑F) and TEG‑NH_2_ to MG‑DAE‑I_TEG_ (G‑I) with subsequent blue light locking.

Table S4. Diameter of MG‑DAE‑I_x_, MG‑DAE‑I_Oct_, and MG‑DAE‑I_TEG_ microgels determined *via* BFSTEM images.

| Sample | Diameter (nm) |
| --- | --- |
| MG-DAE‑I_x_ 1 mol% | 272.0 ± 26.4 |
| MG-DAE‑I_x_ 2 mol% | 155.7 ± 14.9 |
| MG-DAE‑I_x_ 3 mol% | 112.2 ± 12.3 |
| MG-DAE-I_Oct_ 1 mol% | 222.7 ± 18.5 |
| MG-DAE-I_Oct_ 2 mol% | 161.7 ± 9.3 |
| MG-DAE-I_Oct_ 3 mol% | 125.8 ± 11.5 |
| MG-DAE-I_TEG_ 1 mol% | 206.2 ± 15.5 |
| MG-DAE-I_TEG_ 2 mol% | 173.7 ± 14.9 |
| MG-DAE-I_TEG_ 3 mol% | 120.5 ± 17.7 |

Responsivity of DAE-Crosslinked Microgels


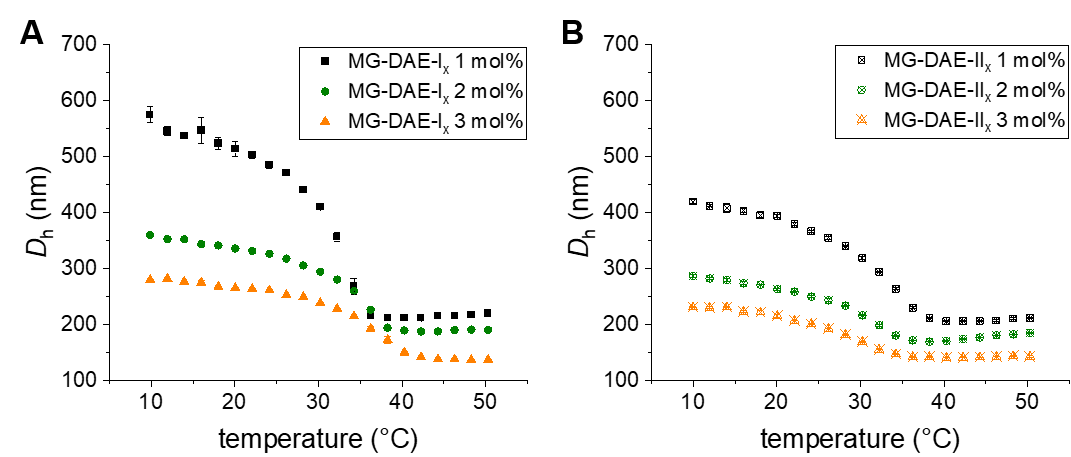


Figure S4. Cooling curves of the MG-DAE-I_x_ (A) and MG-DAE-II_x_ (B) microgels in HPLC water from 10 – 50 °C determined *via* DLS.


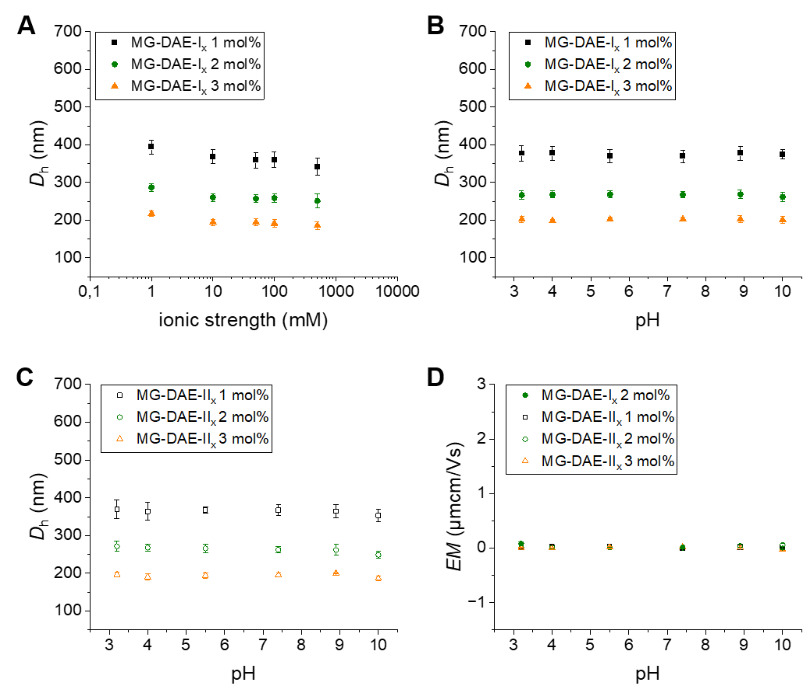


Figure S5. DLS measurements of the MG-DAE-I_x_ microgels in dependence on the ionic strength (A) and pH (B) at 20 °C. C) The pH responsivity of the MG-DAE-II_x_ microgels at 20 °C. D) Electrophoretic mobility of the MG-DAE-II_x_ microgels and representative of MG-DAE-I_x_ 2 mol% in the different buffered solutions at 20 °C.

Table S5. Hydrodynamic diameters and PDI values of the MG-DAE‑I_x_ microgels in dependence on pH values at 50 °C determined *via* DLS.

| pH | MG-DAE‑I_x_ 1 mol% | | MG-DAE‑I_x_ 2 mol% | | MG-DAE‑I_x_ 3 mol% | |
| --- | --- | --- | --- | --- | --- | --- |
|  | *D*_h_ (nm) | PDI (a.u.) | *D*_h_ (nm) | PDI (a.u.) | *D*_h_ (nm) | PDI (a.u.) |
| 3.2 | 259.7 ± 1.4 | 0.036 | 230.5 ± 0.3 | 0.042 | 122.2 ± 0.9 | 0.046 |
| 4.0 | 290.7 ± 1.3 | 0.012 | 259.2 ± 1.0 | 0.034 | 129.7 ± 0.5 | 0.013 |
| 5.5 | 332.6 ± 15.0 | 0.073 | 287.3 ± 11.9 | 0.010 | 182.7 ± 4.0 | 0.099 |
| 7.4 | 335.3 ± 9.4 | 0.052 | 321.7 ± 11.4 | 0.011 | 180.0 ± 3.2 | 0.066 |
| 8.9 | 454.1 ± 71.3 | 0.023 | 463.7 ± 65.3 | 0.124 | 261.7 ± 4.0 | 0.054 |
| 10.0 | 495.3 ± 88.3 | 0.014 | 570.6 ± 102.2 | 0.033 | 282.0 ± 12.0 | 0.041 |

Table S6. Hydrodynamic diameters and PDI values of the MG-DAE‑II_x_ microgels in dependence on pH values at 50 °C determined *via* DLS.

| pH | MG-DAE‑II_x_ 1 mol% | | MG-DAE‑II_x_ 2 mol% | | MG-DAE‑II_x_ 3 mol% | |
| --- | --- | --- | --- | --- | --- | --- |
|  | *D*_h_ (nm) | PDI (a.u.) | *D*_h_ (nm) | PDI (a.u.) | *D*_h_ (nm) | PDI (a.u.) |
| 3.2 | 233.2 ± 1.5 | 0.146 | 183.0 ± 0.6 | 0.074 | 128.4 ± 3.2 | 0.140 |
| 4.0 | 246.8 ± 2.1 | 0.008 | 187.8 ± 3.0 | 0.061 | 133.8 ± 11.3 | 0.131 |
| 5.5 | 316.5 ± 9.8 | 0.043 | 268.1 ± 1.5 | 0.033 | 150.0 ± 0.6 | 0.087 |
| 7.4 | 346.0 ± 27.6 | 0.052 | 397.6 ± 32.7 | 0.048 | 246.1 ± 5.0 | 0.066 |
| 8.9 | 570.2 ± 96.2 | 0.467 | 491.8 ± 87.2 | 0.109 | 520.8 ± 80.5 | 0.117 |
| 10.0 | 504.5 ± 63.6 | 0.059 | 535.5 ± 62.4 | 0.010 | 552.4 ± 70.3 | 0.044 |

Table S7. Hydrodynamic diameters and PDI values of the MG-DAE‑I_x_ microgels at 20 and 50 °C in methanol determined *via* DLS.

| Sample | *D*_h_ 20 °C (nm) | PDI 20 °C (a.u.) | *D*_h_ 50 °C (nm) | PDI 50 °C (a.u.) |
| --- | --- | --- | --- | --- |
| MG-DAE‑I_x_ 1 mol% | 640.1 ± 1.3 | 0.117 | 569.7 ± 9.9 | 0.166 |
| MG-DAE‑I_x_ 2 mol% | 430.9 ± 12.9 | 0.138 | 418.2 ± 7.8 | 0.031 |
| MG-DAE‑I_x_ 3 mol% | 332.4 ± 4.7 | 0.199 | 323.1 ± 3.0 | 0.128 |

Table S8. Hydrodynamic diameters and PDI values of the MG-DAE‑II_x_ microgels at 20 and 50 °C in methanol determined *via* DLS.

| Sample | *D*_h_ 20 °C (nm) | PDI 20 °C (a.u.) | *D*_h_ 50 °C (nm) | PDI 50 °C (a.u.) |
| --- | --- | --- | --- | --- |
| MG-DAE‑II_x_ 1 mol% | 584.5 ± 4.2 | 0.147 | 546.6 ± 7.3 | 0.118 |
| MG-DAE‑II_x_ 2 mol% | 388.7 ± 10.8 | 0.098 | 374.8 ± 5.7 | 0.004 |
| MG-DAE‑II_x_ 3 mol% | 278.6 ± 2.5 | 0.136 | 275.9 ± 13.7 | 0.117 |


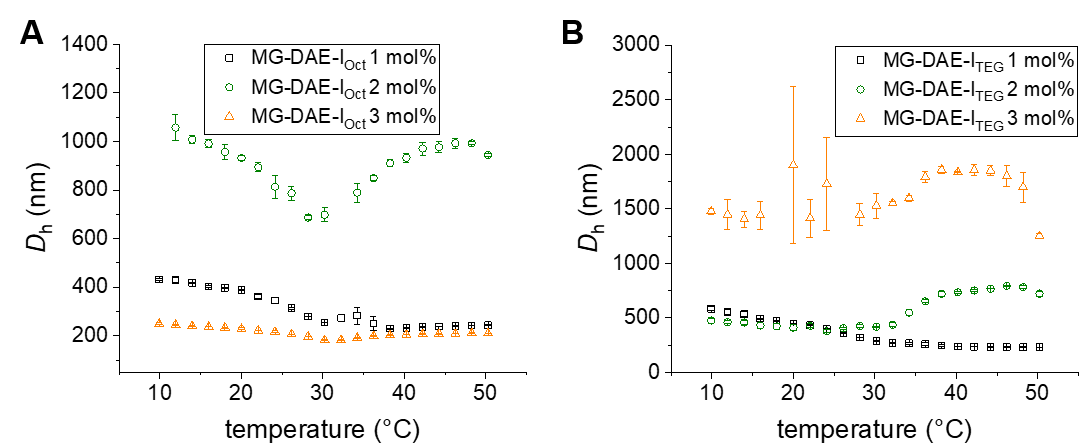


Figure S6. Influence of incorporated hydrophobic (A) and hydrophilic (B) alkyl chains on the temperature-responsivity of the cooling curves in HPLC water determined *via* DLS.

Table S9. Hydrodynamic diameters and PDI values of the MG-DAE‑I_Oct_ microgels at 20 and 50 °C in methanol determined *via* DLS.

| Sample | *D*_h_ 20 °C (nm) | PDI 20 °C (a.u.) | *D*_h_ 50 °C (nm) | PDI 50 °C (a.u.) |
| --- | --- | --- | --- | --- |
| MG-DAE‑I_Oct_ 1 mol% | 541.1 ± 9.4 | 0.118 | 501.0 ± 19.1 | 0.079 |
| MG-DAE‑I_Oct_ 2 mol% | 310.1 ± 7.9 | 0.148 | 295.8 ± 4.2 | 0.058 |
| MG-DAE‑I_Oct_ 3 mol% | 239.9 ± 1.7 | 0.156 | 226.5 ± 1.6 | 0.097 |

Table S10. Hydrodynamic diameters and PDI values of the MG-DAE‑I_TEG_ microgels at 20 and 50 °C in methanol determined *via* DLS.

| Sample | *D*_h_ 20 °C (nm) | PDI 20 °C (a.u.) | *D*_h_ 50 °C (nm) | PDI 50 °C (a.u.) |
| --- | --- | --- | --- | --- |
| MG-DAE‑I_TEG_ 1 mol% | 558.3 ± 9.9 | 0.158 | 494.4 ± 14.2 | 0.322 |
| MG-DAE‑I_TEG_ 2 mol% | 350.5 ± 6.4 | 0.119 | 337.1 ± 4.4 | 0.141 |
| MG-DAE‑I_TEG_ 3 mol% | 242.6 ± 6.3 | 0.180 | 234.6 ± 9.5 | 0.027 |

Photoisomerization in Buffered Media


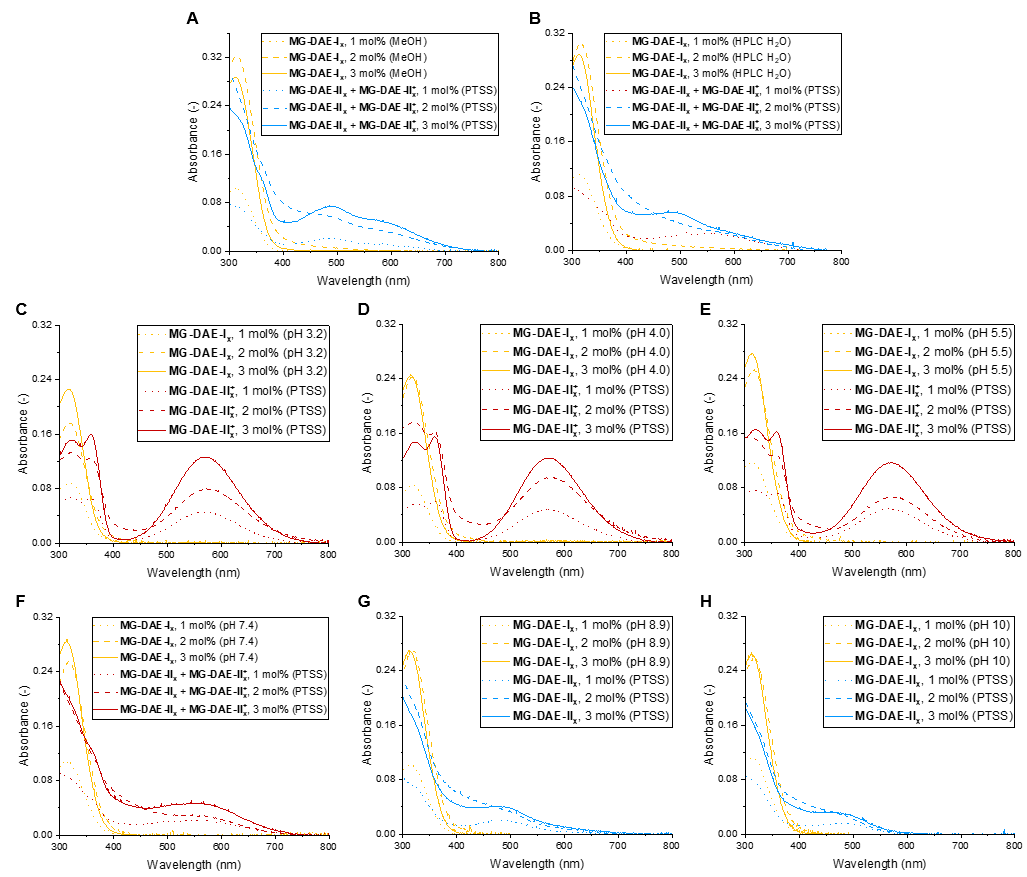


Figure S7. Absorbance spectra of the DAE-crosslinked microgels before irradiation with 365 nm UV light and at the PTSS in A) MeOH, B) HPLC grade water, C) pH 3.2, D) pH 4.0, E) pH 5.5, F) pH 7.4, G) pH 8.9, and H) pH 10 buffers.

Light-Gated Amine Exchange inside the DAE Microgels


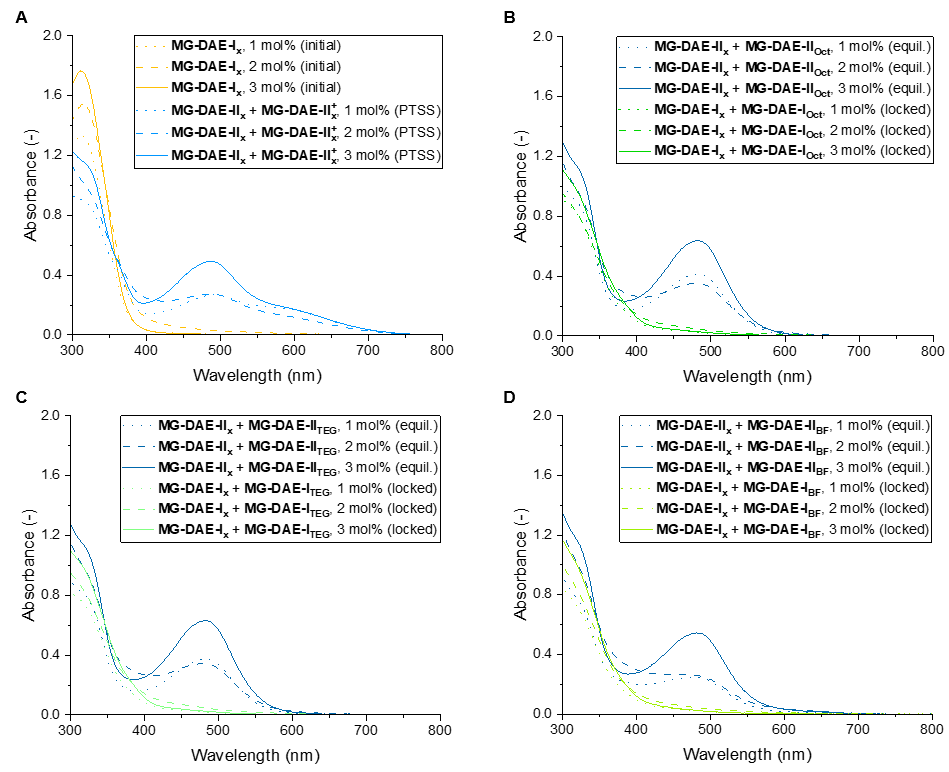


Figure S8. Light-gated amine exchange of the DAE-crosslinked microgels with various primary amines: A) UV/vis spectra of the microgels (~0.5 – 1.0 mg mL^‑1^) before and after UV light irradiation. C) UV/vis spectra of the equilibrated PTSS mixture and Oct‑NH_2_ before and after blue light irradiation. D) UV/vis spectra of the equilibrated PTSS mixture and TEG‑NH_2_ before and after blue light irradiation. E) UV/vis spectra of the equilibrated PTSS mixture and BF‑NH_2_ before and after blue light irradiation.

Blank Experiment with MG‑PVCL


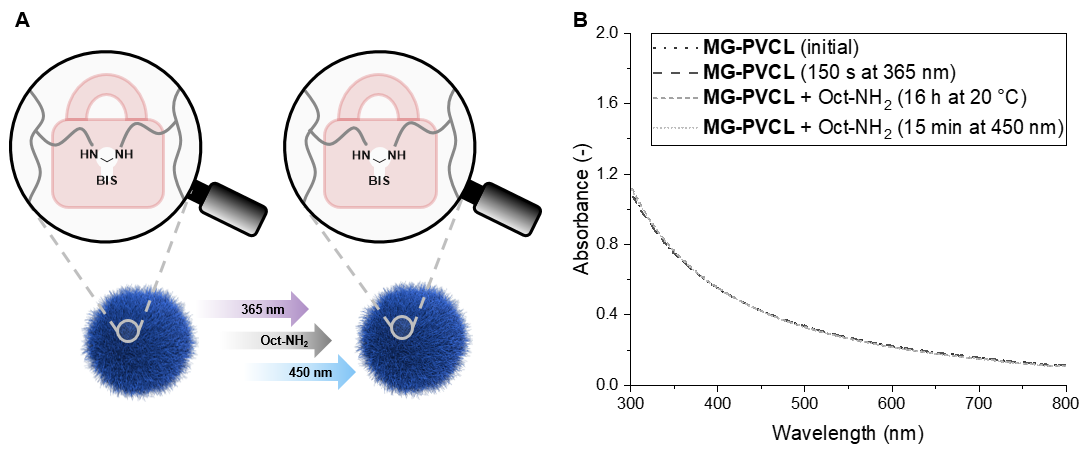


Figure S9. Scattering pattern of MG‑PVCL before and after applying the protocol for light-gated amine exchange revealing no effect of this treatment on the scattering behavior.

Table S11. Comparison of the hydrodynamic diameters and PDI values of MG-PVCL before and after treatment according to the general amine exchange procedure with Oct‑NH_2_ at 20 and 50 °C in water determined *via* DLS.

| Sample | *D*_h_ 20 °C (nm) | PDI 20 °C (a.u.) | *D*_h_ 50 °C (nm) | PDI 50 °C (a.u.) |
| --- | --- | --- | --- | --- |
| MG-PVCL  (before treatment) | 312.8 ± 11.5 | 0.095 | 126.1 ± 3.0 | 0.139 |
| MG-PVCL  (after treatment) | 344.9 ± 17.7 | 0.173 | 146.6 ± 1.8 | 0.220 |

Light-Gated Release of BF‑NH_2_


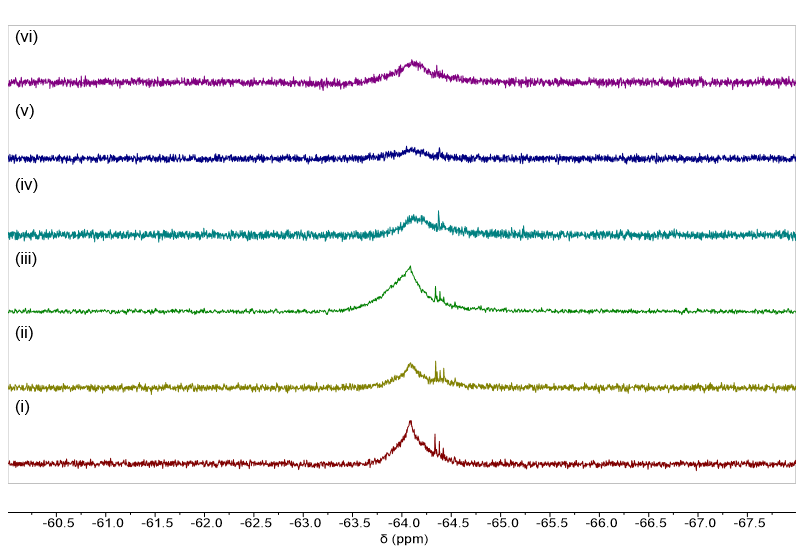


Figure S10. ^19^F{^1^H} NMR spectra of the BF‑NH_2_ incorporated microgels containing 1 (i, iv), 2 (ii, v), and 3 mol% (iii, vi) DAE before (i – iii) and after (iv – vi) light-gated release of BF‑NH_2_, showing a decrease of the ^19^F signal intensities.

NMR Spectra


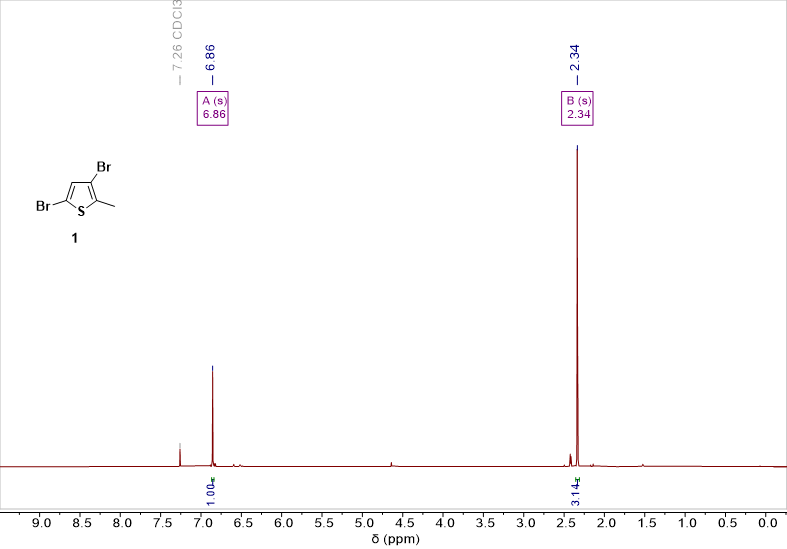


Figure S11. ^1^H NMR spectrum of thiophene 1 in CDCl_3_.


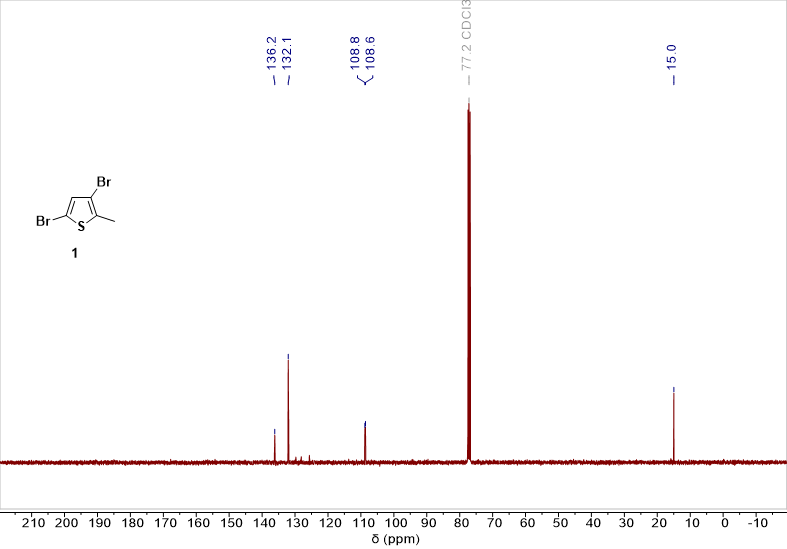


**Figure S12.** ^13^C{^1^H} NMR spectrum of thiophene **1** in CDCl_3_.


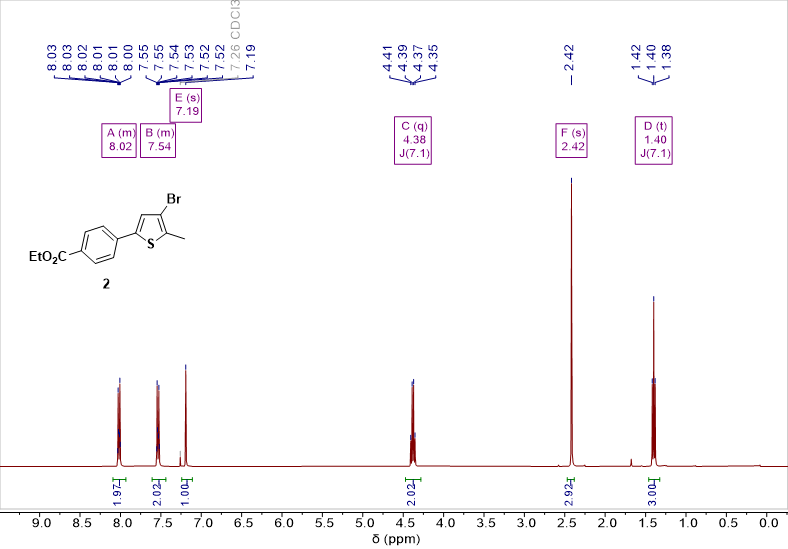


Figure S13. ^1^H NMR spectrum of thiophene 2 in CDCl_3_.


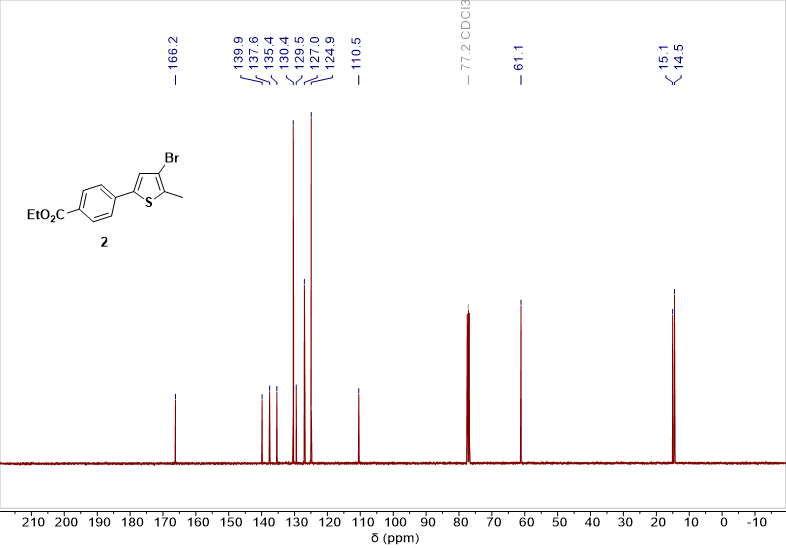


**Figure S14.** ^13^C{^1^H} NMR spectrum of thiophene **2** in CDCl_3_.


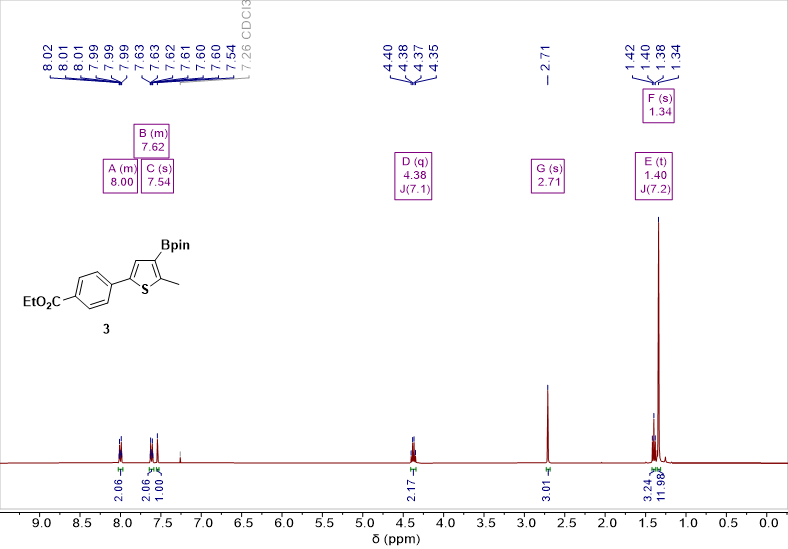


Figure S15. ^1^H NMR spectrum of boronic ester 3 in CDCl_3_.


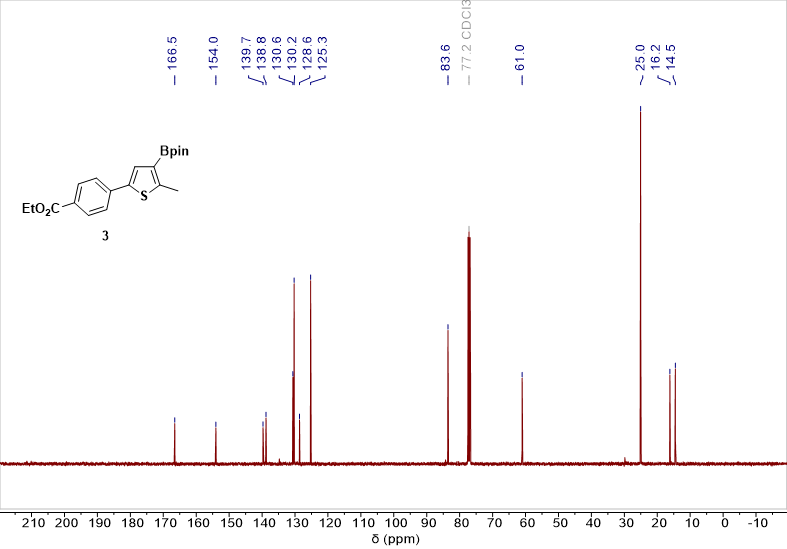


Figure S16. ^13^C{^1^H} NMR spectrum of boronic ester 3 in CDCl_3_.


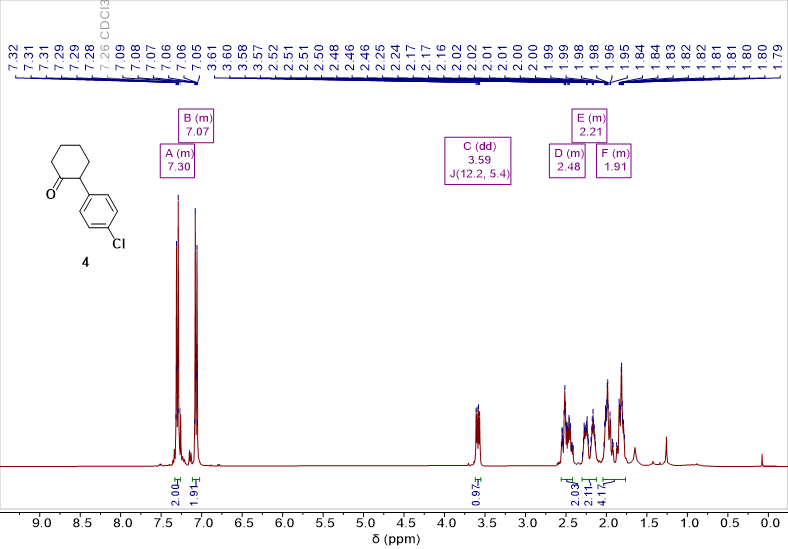


Figure S17. ^1^H NMR spectrum of ketone 4 in CDCl_3_.


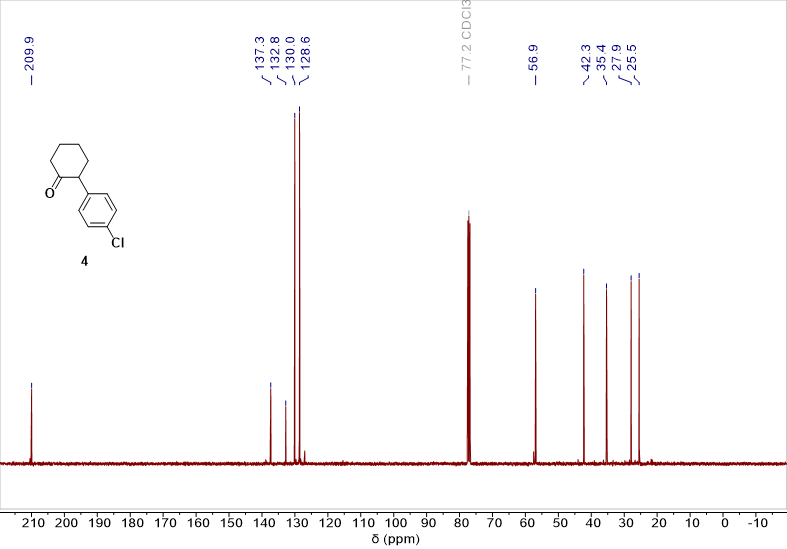


Figure S18. ^13^C{^1^H} NMR spectrum of ketone 4 in CDCl_3_.


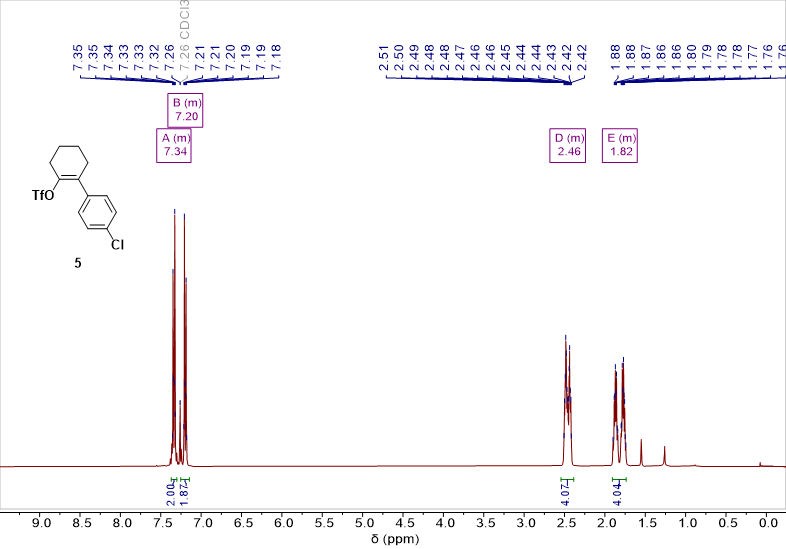


**Figure S19.** ^1^H NMR spectrum of triflate **5** in CDCl_3_.


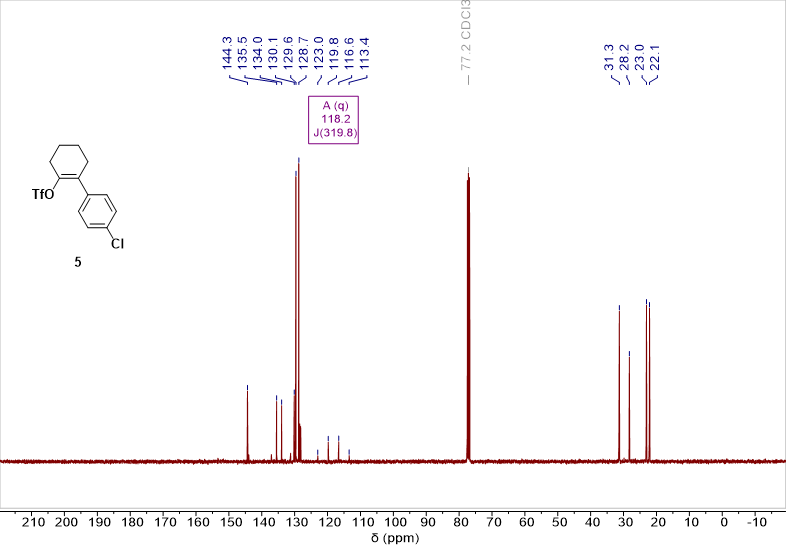


Figure S20. ^13^C{^1^H} NMR spectrum of triflate 5 in CDCl_3_.


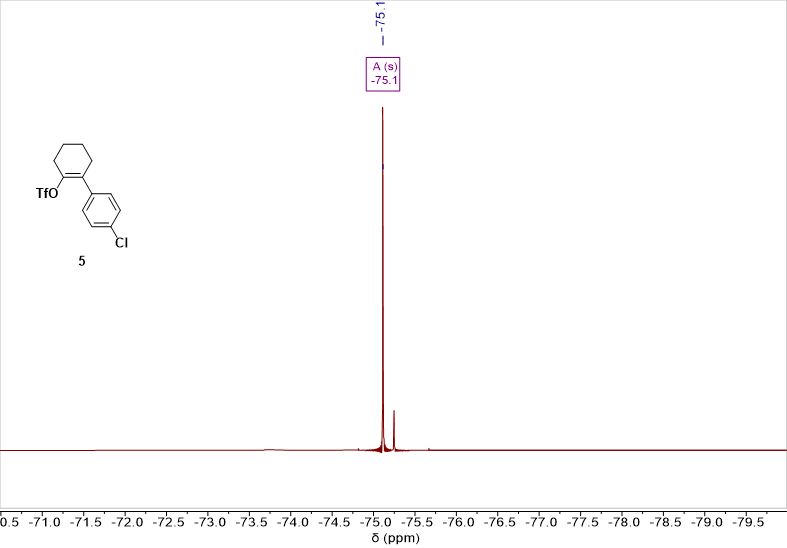


Figure S21. ^19^F{^1^H} NMR spectrum of triflate 5 in CDCl_3_.


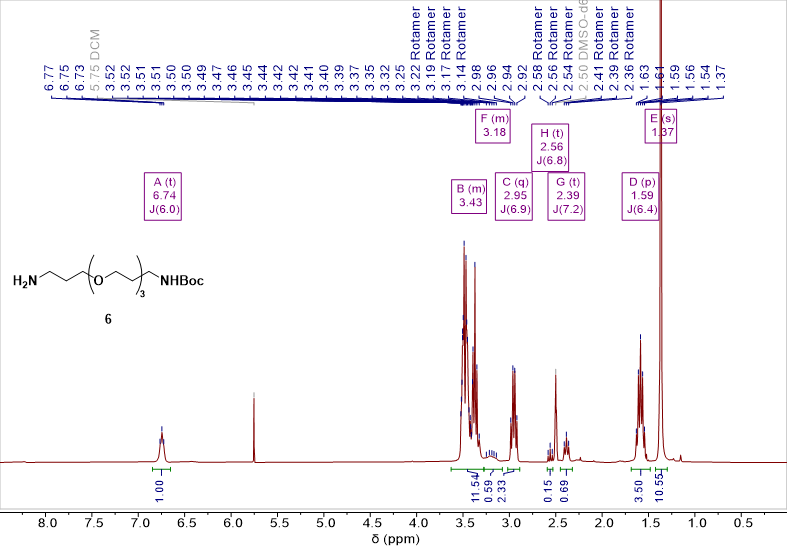


Figure S22. ^1^H NMR spectrum of Boc-protected amine 6 in DMSO‑*d*_6_.


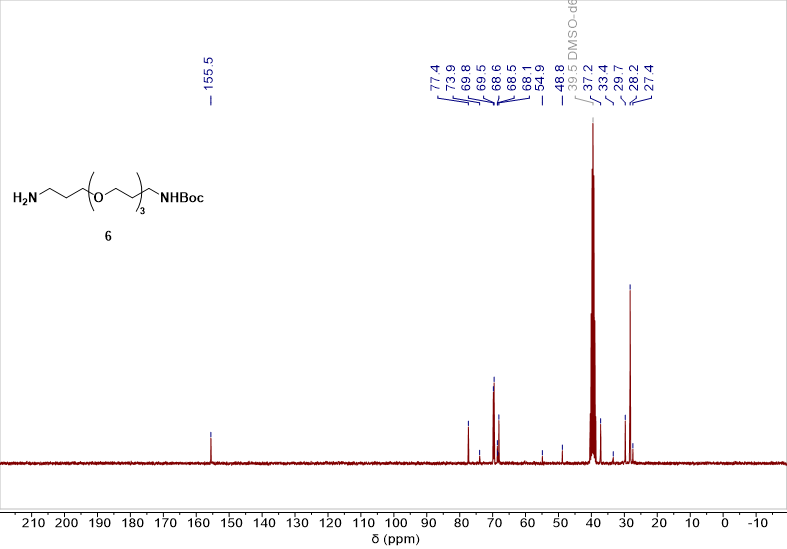


Figure S23. ^13^C{^1^H} NMR spectrum of Boc-protected amine 6 in DMSO‑*d*_6_.


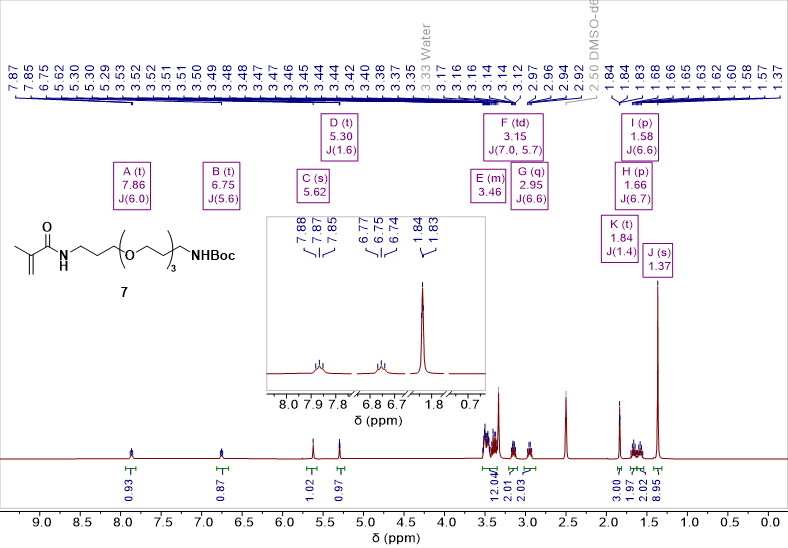


Figure S24. ^1^H NMR spectrum of methacrylamide 7 in DMSO-*d*_6_.


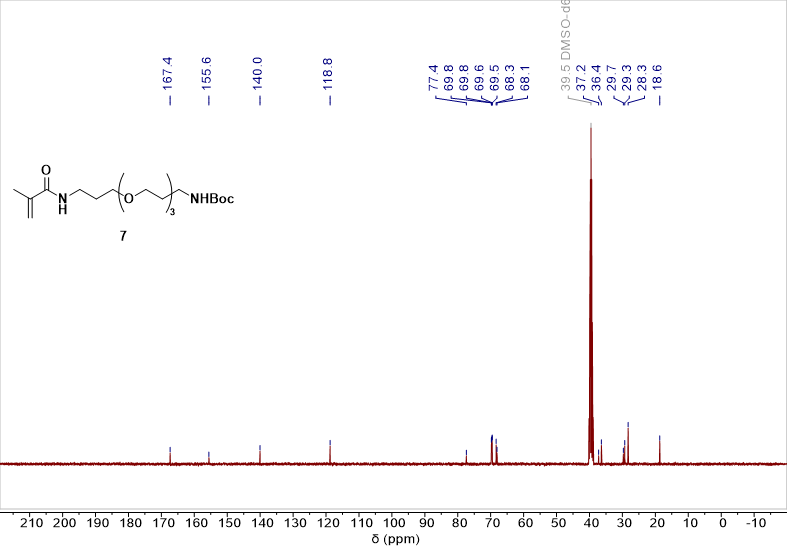


Figure S25. ^13^C{^1^H} NMR spectrum of methacrylamide 7 in DMSO‑*d*_6_.


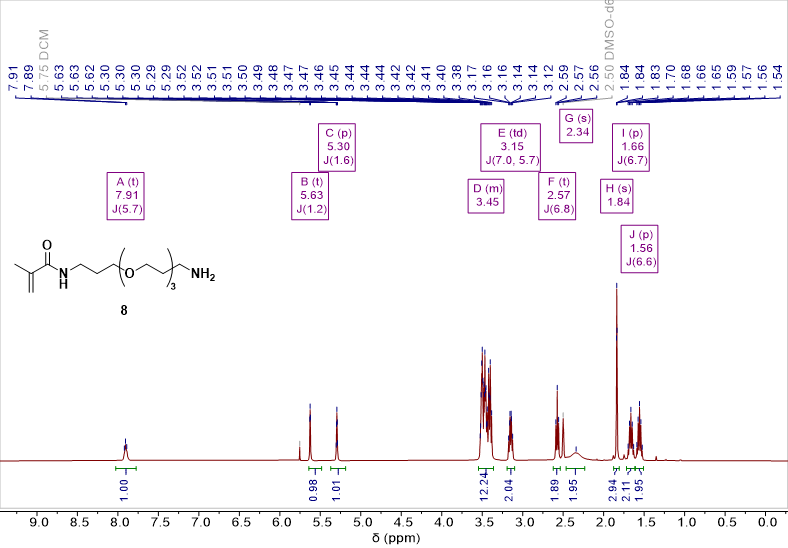


Figure S26. ^1^H NMR spectrum of amine 8 in DMSO‑*d*_6_.


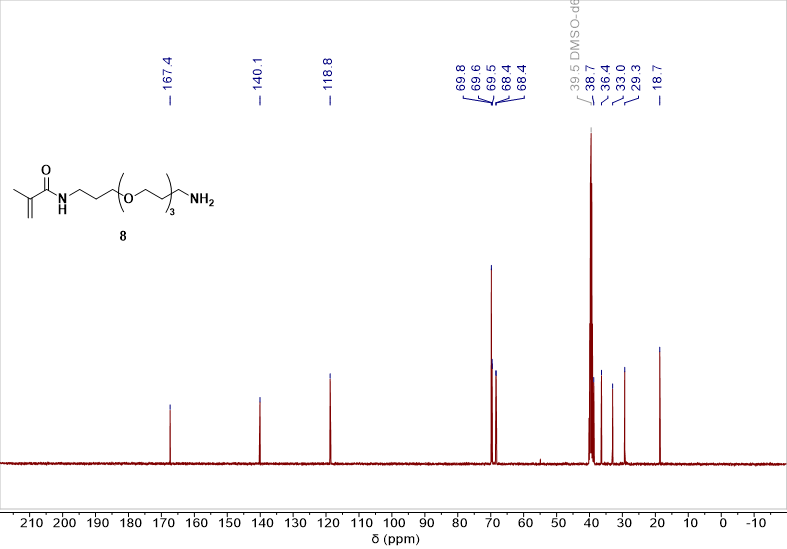


Figure S27. ^13^C{^1^H} NMR spectrum of amine 8 in DMSO‑*d*_6_.


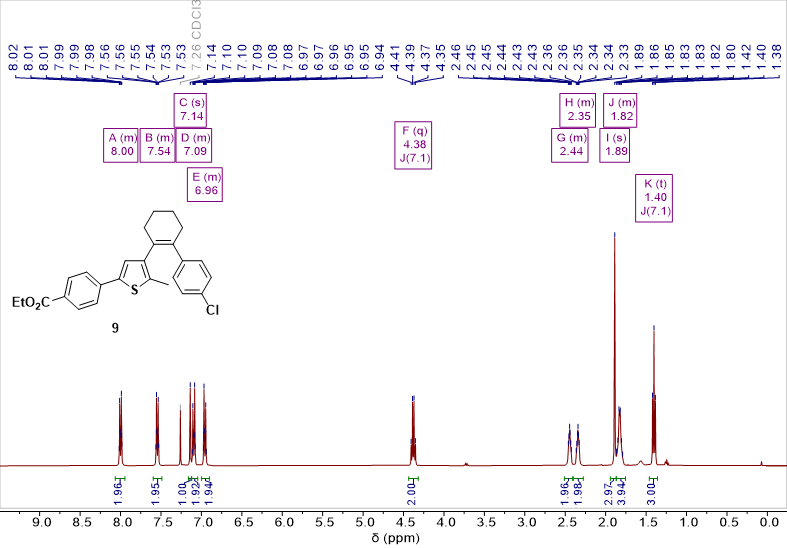


**Figure S28.** ^1^H NMR spectrum of benzoate **9** in CDCl_3_.


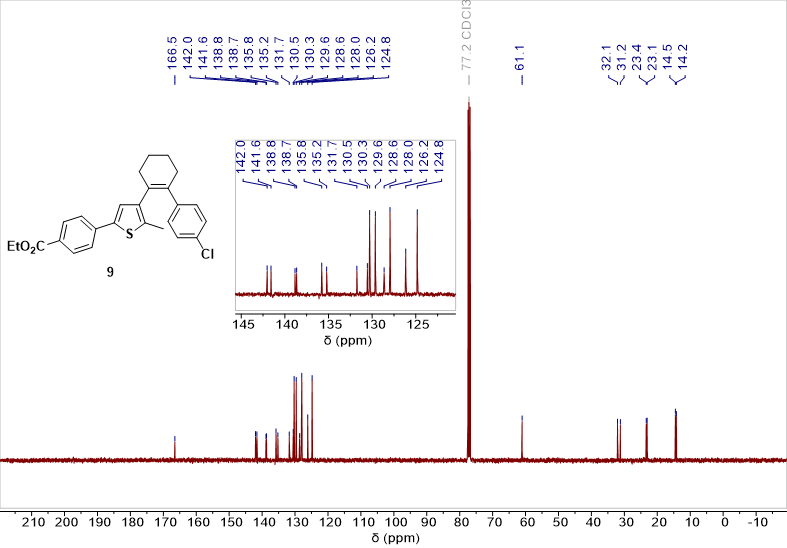


Figure S29. ^13^C{^1^H} NMR spectrum of benzoate 9 in CDCl_3_.


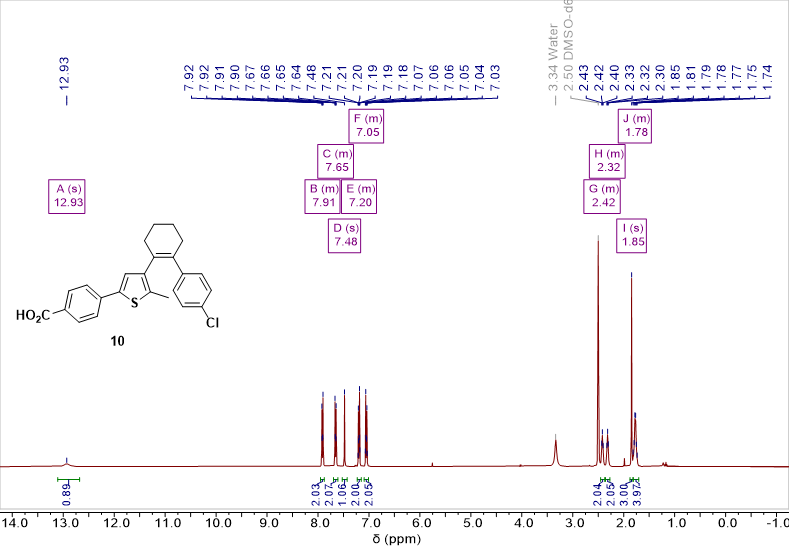


Figure S30. ^1^H NMR spectrum of benzoic acid 10 in DMSO‑*d*_6_.


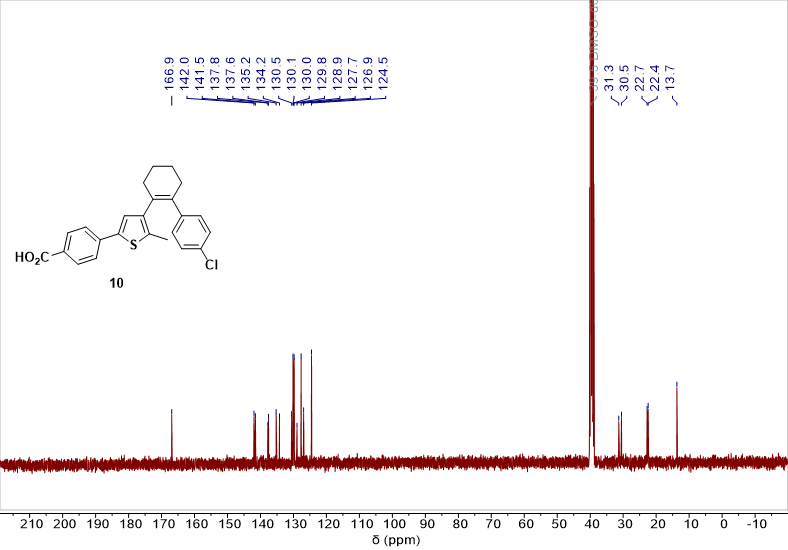


Figure S31. ^13^C{^1^H} NMR spectrum of benzoic acid 10 in DMSO‑*d*_6_.


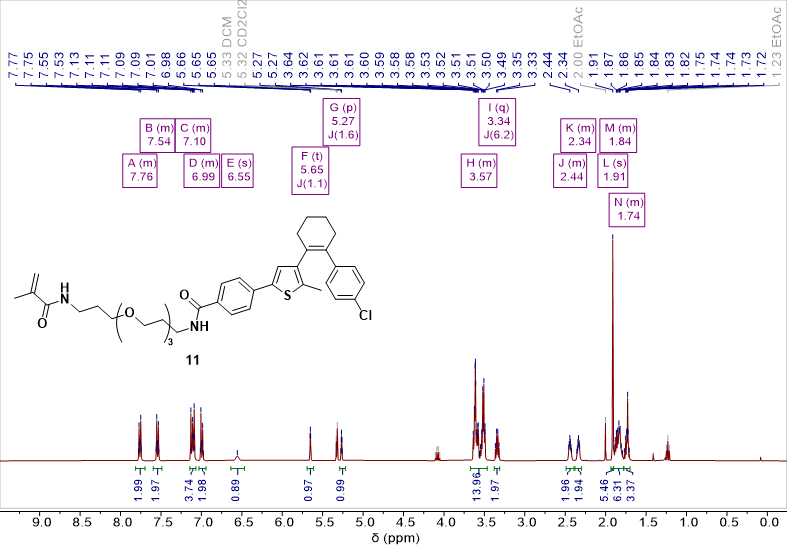


Figure S32. ^1^H NMR spectrum of chlorobenzene 11 in CD_2_Cl_2_.


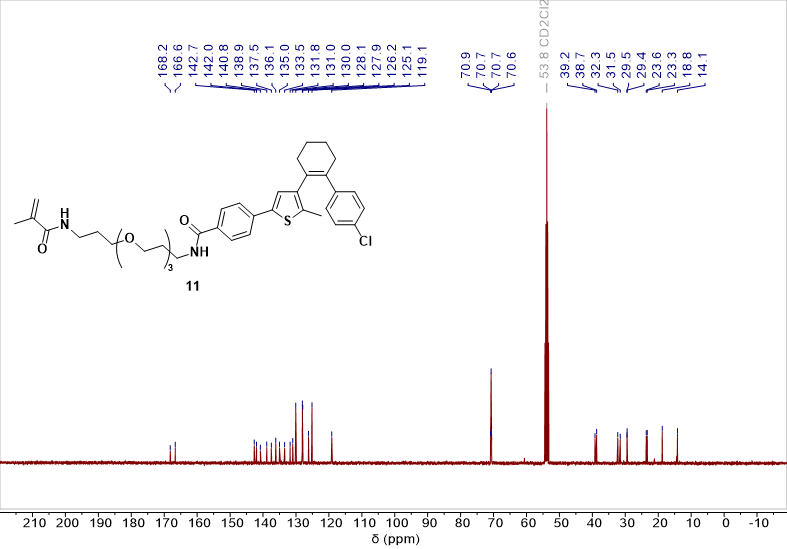


Figure S33. ^13^C{^1^H} NMR spectrum of chlorobenzene 11 in CD_2_Cl_2_.


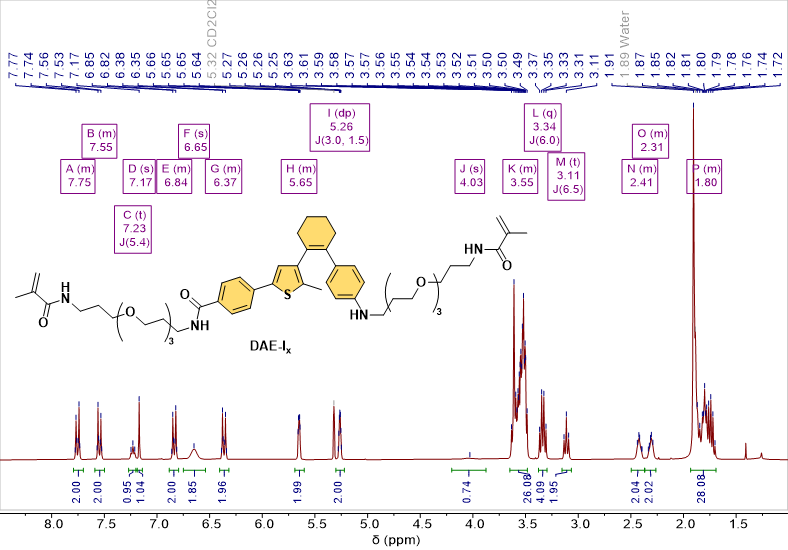


**Figure S34.** ^1^H NMR spectrum of crosslinker **DAE‑I_x_** in CD_2_Cl_2_.


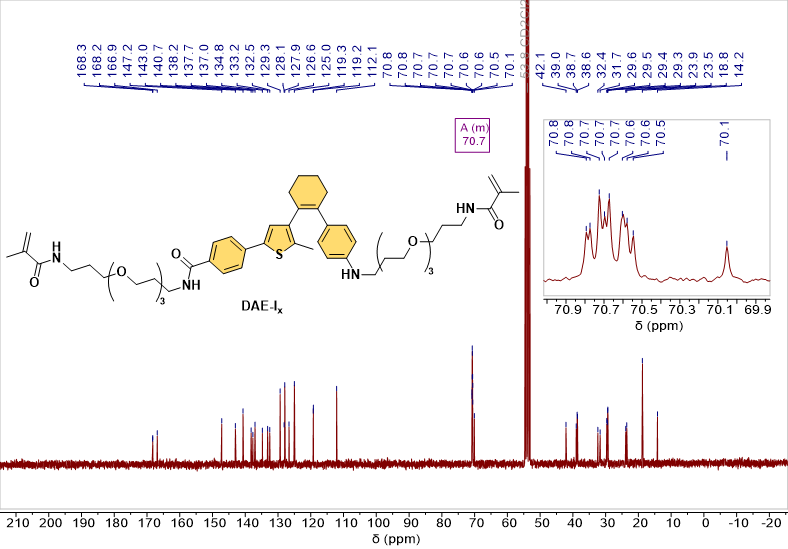


**Figure S35.** ^13^C{^1^H} NMR spectrum of crosslinker **DAE‑I_x_** in CD_2_Cl_2_.

References

[1] D. D. Perrin, *Aust. J. Chem.* **1963**, *16*, 572-578.

[2] V. Valderrey, A. Bonasera, S. Fredrich, S. Hecht, *Angew. Chem. Int. Ed.* **2017**, *56*, 1914-1918.

[3] S. Fredrich, A. Bonasera, V. Valderrey, S. Hecht, *J. Am. Chem. Soc.* **2018**, *140*, 6432-6440.

[4] S. Nagorny, F. Lederle, V. Udachin, T. Weingartz, E. G. Hübner, S. Dahle, W. Maus-Friedrichs, J. Adams, A. Schmidt, *Eur. J. Org. Chem.* **2021**, *2021*, 3178-3189.

[5] J. Zhu, Y. Chen, Y. Geng, H. Cao, S. Li, Z. Li, Q. Wang, T. Su, C. Fu, Shanghai Patent and Trademark Office, CN Patent 113234036A, China, **2021**.

[6] T. Miyoshi, T. Miyakawa, M. Ueda, O. Miyata, *Angew. Chem. Int. Ed.* **2011**, *50*, 928-931.

[7] F. Eisenreich, M. Kathan, A. Dallmann, S. P. Ihrig, T. Schwaar, B. M. Schmidt, S. Hecht, *Nat. Catal.* **2018**, *1*, 516-522.

[8] M. Kathan, F. Eisenreich, C. Jurissek, A. Dallmann, J. Gurke, S. Hecht, *Nat. Chem.* **2018**, *10*, 1031-1036.

[9] L. Zhang, Y. Wu, L. Brunsveld, *Angew. Chem. Int. Ed.* **2007**, *46*, 1798-1802.

[10] Z. P. Gates, B. Dhayalan, S. B. H. Kent, *Chem. Commun.* **2016**, *52*, 13979-13982.

[11] D. Kalsi, N. Barsu, B. Sundararaju, *Chem. Eur. J.* **2018**, *24*, 2360-2364.

[12] E. B. da Silva, D. A. Rocha, I. S. Fortes, W. Yang, L. Monti, J. L. Siqueira-Neto, C. R. Caffrey, J. McKerrow, S. F. Andrade, R. S. Ferreira, *J. Med. Chem.* **2021**, *64*, 13054-13071.

[13] S. M. King, S. L. Buchwald, *Org. Lett.* **2016**, *18*, 4128-4131.

[14] A. Nikolopulos R. Luther, *Z. Phys. Chem.* **1913**, *82U*, 361-384.

[15] A. Beer, *Grundriss des photometrischen Calcüles*, Vieweg, Braunschweig, **1854**.
